# Sleep patterns, sociodemographic correlates, and their association with economic preferences among Indian smallholder farmers

**DOI:** 10.1038/s41598-025-06482-z

**Published:** 2025-07-16

**Authors:** Hao Luo, Selina Bruns, Oliver Musshoff, Daniel Hermann

**Affiliations:** 1 Department of Agricultural Economics and Rural Development, University of Göttingen, D-37073 Göttingen, Germany; 2 University of Bristol, Bristol, BS8 1QU UK; 3 University of Bonn, D-53115 Bonn, Germany

**Keywords:** Sleep patterns, Risk, time and social preferences, Field data, Non-WEIRD populations, Human behaviour, Health policy, Psychology and behaviour

## Abstract

Sleep considerably influences a wide range of daily decisions and activities, yet sleep research largely focuses on well-off populations or relies on controlled laboratory settings. This study provides a field-based perspective on sleep patterns and their relationship with economic preferences, using wrist-measured sleep data from 268 smallholder farmers near Bengaluru, India. We find that participants have relatively high sleep quality, with an average total sleep duration of 6.9 hours per day, including 6.4 hours of nighttime sleep, and 47% exhibiting daytime sleep habits. Sleep patterns vary by sociodemographic factors: older individuals sleep less, while females sleep longer and more closely align with the recommended seven hours of nighttime sleep than males. Higher socioeconomic status correlates with better sleep quality. Additionally, sleep behaviors are linked to individual risk and time preferences, though no clear statistically significant relationship is found with social preferences. Age and gender differences further shape these associations, highlighting important heterogeneity in the data. These findings contribute to a broader understanding of sleep and its correlations with economic preferences in rural contexts, with field sleep data providing a more accurate reflection of natural sleep patterns compared to laboratory or self-reported data alone.

## Introduction

Sleep, a fundamental component of human life, has been shown to have critical relevance to individual economic preferences, decision-making, and behavior^1–3^. However, much of the evidence on sleep comes from controlled laboratory studies or well-off populations, which may not reflect natural sleep patterns, underscoring the need for real-world data^4^. In developing countries, knowledge on sleep patterns is often based on subjective assessments. A review study of sleep parameters in low- and middle-income countries (LMICs) finds that only two out of 45 studies utilized objective measures like polysomnography (PSG) or actigraphy^5^. This gap highlights the need for deeper insights into sleep behaviors through objective measures in the field, particularly for non-WEIRD (Western, Educated, Industrialized, Rich, and Democratic) populations, as emphasized by Rao et al. (2021)^6^. Poorer populations are more likely to suffer from poor sleep, a relationship that remains statistically significant even after controlling for other factors^7^. Sleep patterns may contribute to economic outcome heterogeneity among the rural poor, and understanding their association with economic preferences could reveal previously overlooked avenues for improving welfare and reducing poverty. Yet, field-based economic studies with objectively measured sleep data remain scarce, particularly in LMICs.

To contribute empirical insights from a non-WEIRD population, our objective is to provide a comprehensive description of sleep patterns observed under natural conditions outside of laboratory settings, while examining how individual demographic and socioeconomic factors are correlated with these patterns. Additionally, we investigate the relationship between sleep behaviors and individual core economic preferences, including risk, time, and social preferences. We focus on smallholder farm households, a group highly vulnerable to poverty. Smallholder farmers represent a considerable portion of the global poor, underscoring their critical role in achieving the Sustainable Development Goals^8^. Given the unique nature of their livelihoods, both the sleep patterns and their relevance to the economic preferences of smallholder farmers are likely to differ from those of urban populations. On the one hand, responsibilities such as milking cows or feeding animals at specific times of the day, coupled with the demands of physical labor, can disrupt sleep schedules. This is particularly true for tasks that are too intense to perform during the hot midday hours, especially among farmers with limited access to machinery. On the other hand, rural environments may offer advantages such as reduced exposure to common barriers to sleep, like noise or light pollution, potentially leading to better sleep quality.

We collected field data from 268 smallholder farm households located at the rural-urban interface of Bengaluru, India, between April and July 2022. The data cover individual demographic and socioeconomic characteristics, sleep duration and quality, as well as individual core economic preferences, including risk, time, and social preferences. Respondents first completed a survey to provide sociodemographic information about themselves and their households, along with self-assessed sleep behaviors, receiving a monetary reward of 150 INR (approximately 1.80 USD) upon completion. They were then invited to voluntarily wear a smartwatch for up to seven days, with full information on the device’s functionality and assurances that data would remain anonymous. An additional incentive of 100 INR (approximately 1.20 USD) was offered for smartwatch participation, and enumerators returned to the household within seven days to retrieve the devices. After the smartwatch collection, subjects were asked to voluntarily participate in several incentivized economic tasks, with each task’s final payoff based on individual decisions. Risk preference is elicited using the Eckel and Grossman (EG) task^9^ and time preference is assessed by the Coller and Williams (CW) task^10^. Social preference, specifically aversion to advantageous inequality, is evaluated using a modified Dictator Game (DG)^11^. The average payoffs are 251 INR (about 3.00 USD) for risk, 123 INR (about 1.47 USD) for time, and 79 INR (about 0.95 USD) for social preference tasks. On average, participants earn a total of 703 INR (approximately 8.45 USD) from participating in these parts of the study, slightly exceeding the sample’s average daily personal income of 592.9 INR (approximately 7.11 USD). Supplementary Note 1 provides detailed instructions for the entire data collection process.

## Results

### Descriptive statistics

Table 1 presents the descriptive overview of the sample, featuring individual demographic and socioeconomic characteristics in Panel A, sleep quantity and quality measures, assessed through both wrist-based and self-reported data, in Panel B, and individual preference parameters in Panel C.

**Table 1:** Descriptive statistics summary. Notes: The column *n* represents the number of individuals. *Social status comparison* is a self-assessment of household social standing on a scale from 1 (low), 2 (average), to 3 (high). *Living area* is categorized from 1 (rural), 2 (semi-rural), to 3 (urban). *Daytime sleep frequency*, *daytime sleep duration*, and *Nap frequency per day* include only individuals who get daytime sleep. *Sleep duration per night* was originally measured in hours and is presented in minutes for consistency. Individual time and social preferences are based on participants who made consistent decisions in each task, and only these participants are included in the following analyses. For risk preference, the full sample is used in all analyses, as the task requires a single choice from a set of nine lotteries, which does not allow for inconsistencies in responses.

Variable	Mean	Std. dev.	Min	Max	n
*Panel A. Individual demographic and socioeconomic characteristics*					
Age (in years)	48.183	13.591	19	90	268
Female	0.519	–	0	1	268
Married	0.851	–	0	1	268
Religion (Hindu)	0.977	–	0	1	266
Caste (General)	0.521	–	0	1	267
Household size	4.811	2.546	1	22	264
Education (in years)	6.373	4.830	0	17	268
Monthly income (in 1,000 INR)	11.858	11.205	0	100	265
Social status comparison	2.019	0.437	1	3	268
Living area	2.711	0.471	1	3	266
*Panel B. Individual sleep patterns*					
Worn days	4.116	1.323	1	7	268
Daily sleep duration (in minutes)	413.672	103.053	74.250	899	268
Sleep quality (1–4 scale)	3.720	0.577	1	4	261
Nighttime sleep duration (in minutes)	385.407	80.323	47	544	264
Insufficient sleep (< 7h; yes = 1, no = 0)	0.652	–	0	1	264
|7–hour sleep deviation| (in minutes)	77.471	60.438	4	373	264
Daytime sleep (yes = 1, no = 0)	0.466	–	0	1	268
Daytime sleep frequency	1.085	0.229	1	2	125
Daytime sleep duration (in minutes)	78.220	69.060	22	394.667	125
*Self-reported survey data*					
Sleep duration per night (in minutes)	439.030	53.469	240	600	268
Sleep quality (1–10 scale)	7.683	1.396	4	10	268
Nap (yes = 1, no = 0)	0.287	–	0	1	268
Nap frequency per day	1	0	1	1	77
*Panel C. Individual economic preferences*					
Risk preference: CRRA	−0.103	0.691	−0.947	1.368	268
Time preference: IDR	2.165	2.316	−0.308	5.977	243
Social preference: Guilt parameter	0.401	0.381	0	1	215

Participants are, on average, 48 years old, with roughly half (51.9%) being women. The majority are married (85.1%) and identify as Hindu (97.7%), with an average household size of around 5 members. Most participants (52%) belong to the General category, not classified under any reserved caste categories (Scheduled Castes, Scheduled Tribes, or Other Backward Classes). The average educational attainment is 6.37 years and the average personal income is around 11,858 INR (about 142.57 USD) per month, aligning closely with the national averages of 6.5 years of schooling^12^ and 153 USD monthly income^13^. Moreover, subjects rated their household’s socioeconomic standing in relation to that of fellow residents within the village, with 1 denoting below-average, 2 representing average, and 3 indicating above-average standing. On average, individuals perceive the social status of their households to align with the village’s average social standing. Based on the rural-urban index^14^, which classifies areas around Bangalore into urban (1), semi-rural (2), and rural (3), our sample has an average index score of 2.71, indicating a tendency towards rural and semi-rural regions.

The average number of days the smartwatches were worn was around 4 days, with a maximum of 7 days. On average, the total daily sleep duration is about 414 minutes (6.9 hours), including 385.5 minutes (6.4 hours) of nighttime sleep. The sleep quality is quite satisfactory, with an average rating of 3.7 on a four-point scale, where a score of four indicates excellent sleep quality. Note that the smartwatch-assessed sleep quality is based on a proprietary, unspecified measure and differs from sleep efficiency, the most commonly used proxy for sleep quality in sleep science, which is defined as the ratio of time asleep to time spent in bed. However, 65.2% of individuals suffer from insufficient sleep duration, averaging less than seven hours of sleep per night. Furthermore, 125 individuals (46.6%) exhibit a habit of daytime sleep. Further details on sleep patterns, categorized into nighttime and daytime periods, will be presented in the following section. In addition to the objectively measured sleep, subjects are asked to self-assess their sleep behavior in the survey. On average, individuals report sleeping 439 minutes (7.3 hours) per night. The self-reported sleep quality averages 7.7 on a ten-point scale, lower than the smartwatch-assessed quality, yet all self-reported ratings remain above 4. Regarding naps, 77 out of 268 subjects (28.7%) report having a habit of taking naps. Among those who nap, all of them report napping only once on a given day. Supplementary Note 2 compares subjective and objective sleep measures and only weak correlations are identified.

**Fig. 1:**
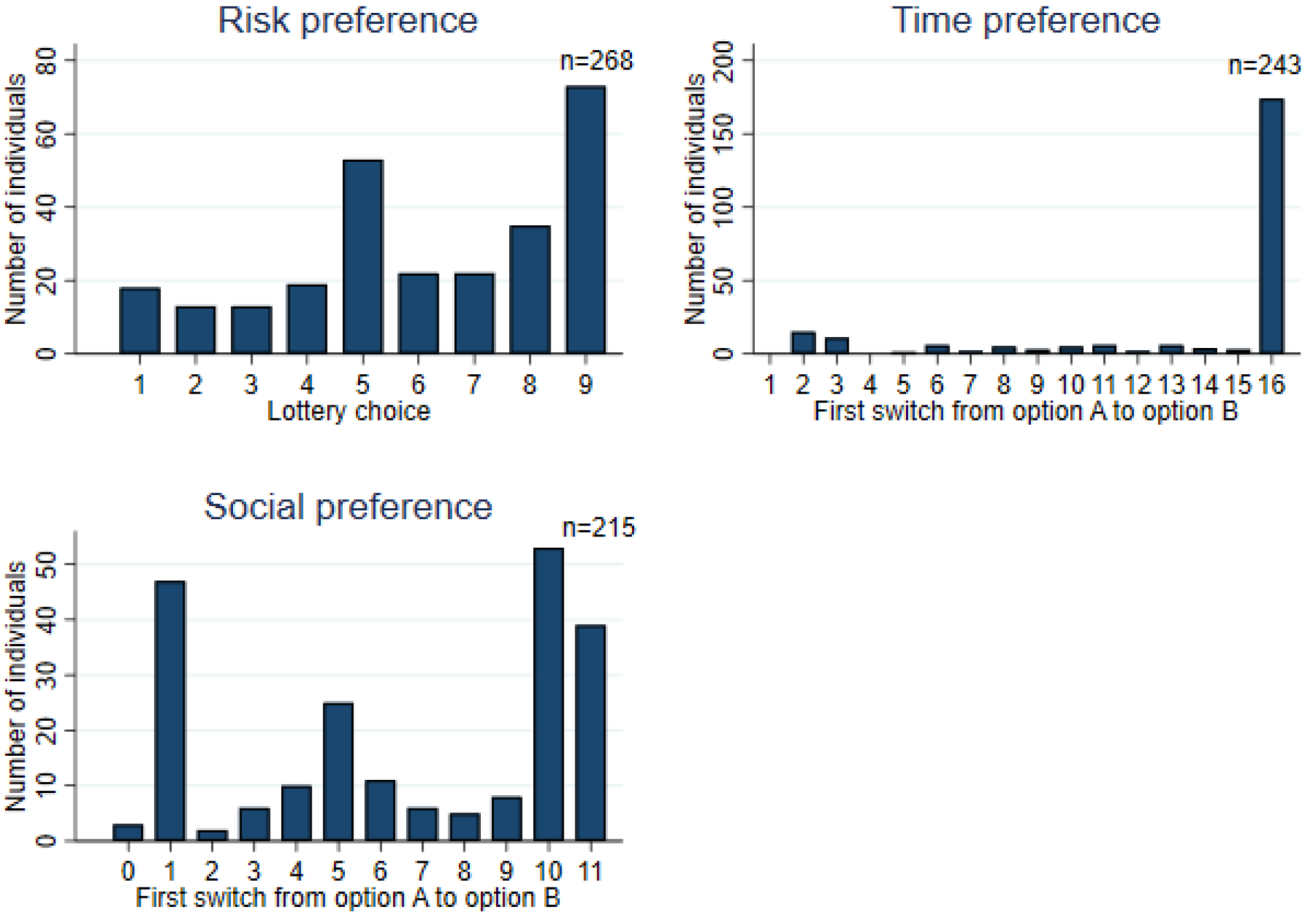
Individual economic preferences. Notes: Risk preference is elicited by the EG task^9^. Individuals selecting lotteries 1-5 are risk-averse, those choosing lottery 6 are risk-neutral, and those choosing lotteries 7-9 are risk-seeking. Time preference is elicited by the CW task^10^ and social preference is elicited by the modified DG^11^. *n* represents the number of individuals.

Individual economic preferences are presented in Panel C of Table 1 and are visualized in Fig. 1. In our sample, the average Constant Relative Risk Aversion (CRRA) coefficient is -0.103, within the risk-neutral range (-0.15, 0.15)^15^. While much of the economic literature suggests that the poor may display a higher level of risk aversion^16^, only 43.28% of participants in our sample are classified as risk-averse. In contrast, 48.51% (130 individuals) exhibit risk-seeking preferences, and the remaining 8.21% (22 participants) are risk-neutral. The results for time and social preferences include only subjects who provided complete and consistent responses in the CW task and the modified DG. After accounting for individual risk aversion, the average individual discounting rate (IDR) is 216.5%. This indicates that the population in this sample is considerably impatient. Notably, more than 70% of participants (174) always chose option A (the top-right panel of Fig. 1), suggesting a greater preference for immediate rewards. Regarding social preference, the average guilt parameter, which indicates the aversion to advantageous inequality, is 0.4. We observe that 18.14% (39 individuals) consistently choose the option with equal payoffs (Option B), yielding a parameter value of 0. This suggests that they are potentially willing to sacrifice more than 1 INR to reduce inequality by 1 INR. Conversely, only three individuals always choose Option A, corresponding to a parameter value of 1, which may indicate a preference for sending money to increase inequality. 56.74% of subjects have a parameter smaller than 0.5, indicating a moderate aversion to advantageous inequality (the bottom-left panel of Fig. 1).

### Sleep patterns

Hereafter, we focus on wrist-based sleep data, providing a detailed description of nighttime and daytime sleep separately.

**Fig. 2:**
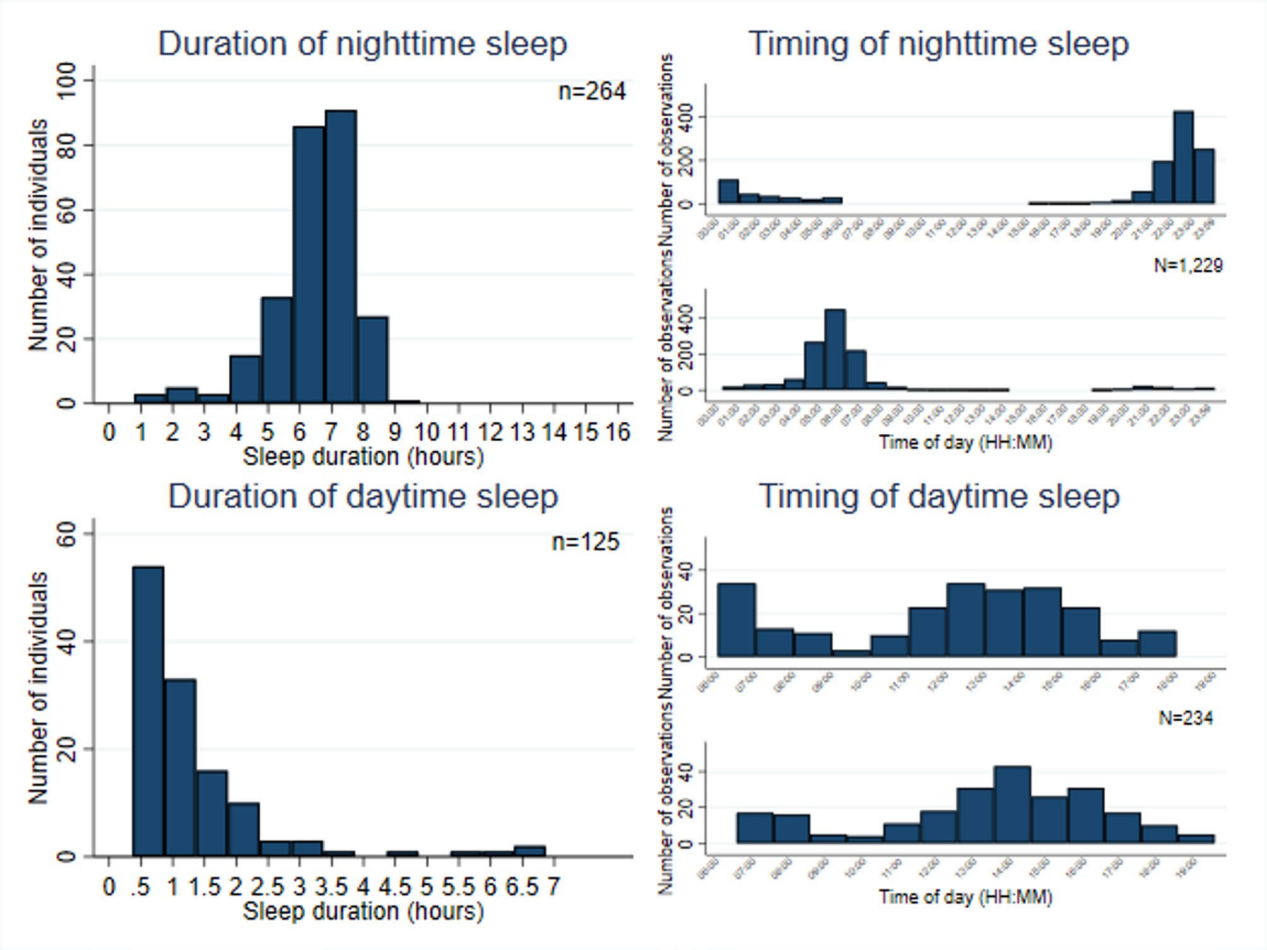
Nighttime and daytime sleep duration and timing. Notes: For timing histograms, the top parts show start times, and the bottom parts show end times for nighttime and daytime sleep. *n* represents the number of individuals and *N* represents the number of observations.

Nighttime sleep holds prominence as the main rest period for most individuals. In our sample, individuals sleep for an average of 6.4 hours (385.4 minutes) per night (Panel B Table 1), slightly below the minimum seven hours recommended by sleep health experts for adults^17^. The top-left panel of Fig. 2 shows that most individuals sleep between six and eight hours at night. While the mean sleep duration is relatively close to the recommendation, 65.2% of individuals sleep less than seven hours per night on average, suggesting a common issue of insufficient sleep. Additionally, a small fraction, 4.92% (13 individuals), average less than 4 hours of sleep per night. To capture the degree to which individuals diverge from the recommended duration of sleep, we computed the 7-hour sleep deviation as the absolute difference between total nightly sleep duration and seven hours. This approach follows Park et al. (2023)^18^ and aligns with evidence suggesting a U-shaped relationship between sleep duration and health outcomes^19^. A higher value indicates a greater absolute departure from the recommended sleep duration. The average absolute 7-hour sleep deviation is 77 minutes (1.3 hours), with deviations ranging from just a few minutes to as much as six hours (Panel B Table 1). Nighttime sleep quality is notably high, averaging 3.7 on a four-point scale, with four representing the highest rating. Another important dimension of sleep behavior is the timing of sleep onset and wake-up. As shown in the top-right panel of Fig. 2, most individuals start their sleep around 11 PM, typically between 9 PM and 12 AM, with a few starting as early as 4 PM, or considerably later. The peak wake-up time is 6 AM, with most participants waking between 5 AM and 7 AM.

Daytime sleep serves as another crucial metric for better understanding sleep patterns among smallholders. Short daytime sleep has been shown to have protective effects on cognitive performance, decision-making, and health status^20–22^. Among the 268 individuals surveyed, 125 (46.64%) sleep during the daytime (Panel B Table 1), with the majority (85.60%) taking only one nap and 18 individuals averaging two naps per day. As illustrated in the bottom-left panel of Fig. 2, the average nap duration of those taking naps is 78 minutes, with a minimum of 22 minutes and a maximum of 395 minutes (6.6 hours). Most naps last between 30 and 90 minutes. Notably, the beneficial effects of daytime sleep do not increase linearly with duration; alertness levels rise with nap length but plateau between 60 and 120 minutes^23^. In our sample, 59 individuals (47.20%) nap for more than 60 minutes, with 21 exceeding 120 minutes, including five who average over four hours. Among those with extended naps exceeding 120 minutes, three individuals do not engage in nighttime sleep. Additionally, for one individual who naps an average of 59 minutes, the data indicate no nighttime sleep recorded. The timing of a nap is considered another factor influencing the benefits of napping. A review study by Dutheil et al. (2021)^20^ indicates that early afternoon naps (before 1 PM) are associated with enhanced cognitive performance. In our sample, most napping takes place in the noon and early afternoon between 11 AM and 4 PM (the bottom-right panel of Fig. 2).

### Heterogenous sleep patterns based on sociodemographic characteristics

Heterogenous sleep patterns concerning various demographic and socioeconomic factors have been extensively documented^24,25^. Table 2 presents correlations between demographic and socioeconomic factors and sleep. Columns (1)-(5) report the results for the entire sample, while columns (6)-(10) focus on individuals with insufficient nighttime sleep (averaging less than seven hours). For both groups, the table displays metrics for overall daily sleep duration and quality, as well as a breakdown of nighttime and daytime sleep.

**Table 2:** Relationship between demographic and socioeconomic characteristics and sleep patterns. Notes: The table presents the results from the mixed-effects regression analysis, including individual random effects. *N* represents the number of observations. Each observation in the regression represents the total sleep aggregated for either the entire day, nighttime, or daytime. Sleep duration is measured in minutes, and sleep quality is rated on a four-point scale, with four indicating the best quality and one the worst. *Social status comparison* is a self-assessment of household social standing on a scale from 1 (low), 2 (average), to 3 (high). *Living area* is categorized from 1 (rural), 2 (semi-rural), to 3 (urban). Column (5) includes only individuals who get daytime sleep and Column (10) includes those who get daytime sleep within the subgroup experiencing insufficient nighttime sleep. *, **, and *** indicate statistical significance at the 0.10, 0.05, and 0.01 level, respectively. Standard errors are shown in parentheses.

	Full sample					Individuals with insufficient sleep (< 7h)				
	Sleep duration per day	Sleep quality	Nighttime sleep duration	|7-hour deviation|	Daytime sleep duration	Sleep duration per day	Sleep quality	Nighttime sleep duration	|7-hour deviation|	Daytime sleep duration
	(1)	(2)	(3)	(4)	(5)	(6)	(7)	(8)	(9)	(10)
Age (in years)	− 7.007***	− 0.003	− 5.701**	− 0.968	− 0.522	− 1.159	0.036	− 0.141	− 1.742	− 6.221
	(2.447)	(0.015)	(2.246)	(1.378)	(3.535)	(2.924)	(0.034)	(2.701)	(2.622)	(6.344)
Age$^2$ (in years)	0.065***	0.000	0.052**	0.011	0.001	0.006	− 0.000	− 0.007	0.023	0.059
	(0.023)	(0.000)	(0.022)	(0.013)	(0.031)	(0.028)	(0.000)	(0.026)	(0.025)	(0.058)
Female	18.620*	0.087	15.680	− 14.631**	8.845	17.220	0.045	19.281*	− 26.603***	3.209
	(11.010)	(0.078)	(9.831)	(7.276)	(13.450)	(11.430)	(0.114)	(10.870)	(10.020)	(17.636)
Married	7.366	0.070	− 1.806	0.641	− 4.657	7.633	0.179	4.858	− 5.118	− 16.772
	(15.970)	(0.102)	(13.360)	(9.480)	(17.451)	(18.240)	(0.155)	(15.930)	(14.220)	(20.200)
Religion (Hindu)	13.890	0.019	− 27.059*	17.620	− 8.325	− 47.414**	0.228	− 18.260	37.550**	34.090*
	(59.660)	(0.151)	(16.020)	(14.910)	(20.596)	(21.680)	(0.249)	(20.930)	(18.680)	(19.566)
Caste (General)	5.052	− 0.019	− 2.544	− 4.914	− 2.844	10.350	0.063	6.622	− 10.510	11.250
	(10.310)	(0.069)	(9.181)	(6.837)	(14.862)	(11.230)	(0.098)	(10.730)	(10.040)	(16.663)
Household size	0.763	− 0.002	− 0.141	− 0.500	− 2.560*	1.792	− 0.002	0.575	− 0.998	− 3.610**
	(1.675)	(0.009)	(1.534)	(1.036)	(1.442)	(1.381)	(0.011)	(1.292)	(1.157)	(1.802)
Education (in years)	0.271	− 0.001	− 0.196	− 0.075	− 0.453	− 0.484	− 0.015	− 1.075	− 0.202	0.197
	(1.395)	(0.009)	(1.272)	(0.933)	(1.698)	(1.509)	(0.012)	(1.350)	(1.230)	(1.593)
Monthly income (in 1,000 INR)	− 0.025	0.004*	− 0.274	0.132	0.436	0.264	0.003	− 0.293	0.176	1.337*
	(0.396)	(0.002)	(0.437)	(0.333)	(0.755)	(0.461)	(0.004)	(0.479)	(0.445)	(0.797)
Social status	0.940	0.150*	− 5.671	6.653	− 2.199	1.347	0.325*	3.319	6.037	− 40.417**
comparison	(13.300)	(0.091)	(12.700)	(9.987)	(12.577)	(17.080)	(0.168)	(17.550)	(16.130)	(16.674)
Living area	0.768	− 0.027	− 6.460	8.514	11.330	4.035	0.024	− 10.820	8.068	29.372**
	(11.380)	(0.072)	(10.190)	(7.475)	(12.270)	(11.880)	(0.103)	(11.520)	(10.460)	(12.742)
Constant	518.311***	3.424***	588.839***	52.000	94.600	397.153***	1.803*	407.301***	70.050	204.631
	(91.113)	(0.503)	(69.195)	(46.357)	(87.468)	(93.320)	(0.982)	(84.384)	(78.607)	(149.619)
N	1,117	959	1,063	1,063	200	715	580	682	682	127

Age and gender are two well-recognized demographic characteristics influencing sleep patterns. Our sample shows a statistically significant relationship between age and sleep duration. Initially, increasing age is associated with a decrease in total daily sleep duration, primarily due to a reduction in nighttime sleep. However, this age-related correlation diminishes after a certain age. Gender differences in total sleep duration and absolute 7-hour sleep deviation are evident. Women exhibit a statistically significant longer daily sleep duration than men, averaging 19 more minutes per day. Additionally, women have a smaller deviation from the recommended minimum of seven hours of nightly sleep, with their average nighttime sleep being approximately 15 minutes closer to this benchmark. Similar statistically significant gender differences are also observed among individuals with insufficient sleep, with women sleeping 19 minutes longer during the nighttime and having a smaller deviation from the recommended seven hours compared with men. The findings suggest that women not only sleep longer than men but also have sleep duration that is more closely aligned with the recommended minimum of seven hours per night, indicating a distinct sleep duration pattern among women in our sample. Regarding other demographic characteristics, household size is statistically significantly negatively correlated with daytime sleep duration in both the full sample and the sample with insufficient sleep.

Socioeconomic status has also been demonstrated to correlate with sleep patterns. In our sample, higher monthly income is statistically significantly associated with better sleep quality. Among individuals with insufficient sleep, higher income is further linked to longer daytime sleep duration. Furthermore, those who perceive their household social standing as higher tend to exhibit better sleep quality. Notably, for individuals with insufficient sleep, a higher perceived social standing is linked to a 40-minute shorter daytime sleep duration.

### Sleep patterns and individual economic preferences

Table 3 represents the relationship between sleep and individual economic preferences, controlling for sociodemographic characteristics. The analyses of time and social preferences are based on data from participants with consistent behavior, while results using the full dataset as a robustness check are presented in Supplementary Table S1, confirming the stability of the estimated coefficients in both sign and magnitude. Given previous findings on age-based and gender-based differences^26,27^, Supplementary Tables S2–S4 display results by these heterogeneities.

**Table 3:** Relationship between sleep patterns and individual economic preferences. Notes: The table presents Ordinary Least Squares regression estimates with individual economic preferences as the dependent variables and various sleep metrics as the explanatory variables, while controlling for individual demographic and socioeconomic characteristics listed in Panel A of Table 1. The results for time and social preferences include only consistent decision-makers in the CW task and the modified DG. *X*$^2$ reports coefficients on the squared sleep duration, accounting for the nonlinear correlations. Sleep duration is measured in minutes, and sleep quality is rated on a four-point scale, with four indicating the best quality and one the worst. *n* represents the number of individuals. *, **, and *** indicate statistical significance at the 0.10, 0.05, and 0.01 level, respectively. Standard errors are shown in parentheses.

	Full sample						Individuals with insufficient sleep (< 7h)					
	X =						X =					
	Sleep duration per day	Sleep quality	Nighttime sleep duration	|7-hour deviation|	Daytime sleep dummy	Daytime sleep duration	Sleep duration per day	Sleep quality	Nighttime sleep duration	|7-hour deviation|	Daytime sleep dummy	Daytime sleep duration
	(1)	(2)	(3)	(4)	(5)	(6)	(7)	(8)	(9)	(10)	(11)	(12)
	*Panel A: Risk preference (CRRA)*											
X	0.000	0.095	− 0.001	0.005**	− 0.031	0.000	− 0.002	0.137*	0.003	0.005**	− 0.082	0.003
	(0.002)	(0.072)	(0.003)	(0.002)	(0.091)	(0.003)	(0.000)	(0.082)	(0.004)	(0.002)	(0.116)	(0.003)
X$^2$	0.000		0.000	− 0.000**		− 0.000	0.000		− 0.000	− 0.000*		− 0.000*
	(0.000)		(0.000)	(0.000)		(0.000)	(0.000)		(0.000)	(0.000)		(0.000)
Constant	− 0.962	− 1.087*	− 0.660	− 1.044*	− 0.822	0.120	0.105	− 0.709	− 0.460	− 0.575	− 0.093	0.226
	(0.767)	(0.650)	(0.797)	(0.593)	(0.598)	(1.116)	(0.996)	(0.755)	(0.957)	(0.714)	(0.730)	(1.241)
*n*	258	251	254	254	258	119	166	161	166	166	166	78

	*Panel B: Time preference (IDR)*											
X	0.007	− 0.082	− 0.003	− 0.010	0.090	− 0.015	0.016**	− 0.191	− 0.016	− 0.014	− 0.134	− 0.021*
	(0.006)	(0.333)	(0.010)	(0.007)	(0.322)	(0.010)	(0.007)	(0.376)	(0.014)	(0.009)	(0.432)	(0.012)
X$^2$	− 0.000		0.000	0.000		0.000	− 0.000**		0.000	0.000		0.000***
	(0.000)		(0.000)	(0.000)		(0.000)	(0.000)		(0.000)	(0.000)		(0.000)
Constant	3.130	3.690	4.107	4.428*	4.118*	3.139	1.105	3.682	5.239	4.644	3.651	1.776
	(2.925)	(2.632)	(2.844)	(2.355)	(2.404)	(3.506)	(3.494)	(3.566)	(3.853)	(3.283)	(3.296)	(4.607)
*n*	234	227	230	230	234	111	148	143	148	148	148	72

	*Panel C: Social preference (guilt parameter)*											
X	− 0.000	− 0.015	− 0.000	0.000	0.046	0.001	− 0.000	0.005	0.000	0.001	0.011	− 0.000
	(0.001)	(0.048)	(0.002)	(0.001)	(0.055)	(0.002)	(0.002)	(0.049)	(0.002)	(0.001)	(0.067)	(0.003)
X$^2$	0.000		0.000	− 0.000		− 0.000	0.000		− 0.000	− 0.000		0.000
	(0.000)		(0.000)	(0.000)		(0.000)	(0.000)		(0.000)	(0.000)		(0.000)
Constant	0.458	0.231	0.368	0.236	0.317	0.039	0.889	0.734	0.905	0.851	0.905	0.631
	(0.497)	(0.429)	(0.500)	(0.425)	(0.409)	(0.587)	(0.683)	(0.566)	(0.670)	(0.591)	(0.567)	(0.697)
*n*	206	201	203	203	206	92	133	129	133	133	133	60

A statistically significant correlation exists between a sleep deviation period of seven hours and the CRRA value. Individuals exhibiting a greater deviation from the recommended seven-hour nighttime sleep duration tend to demonstrate higher levels of risk aversion, however, this correlation becomes negative beyond certain lengths of deviation. This relationship holds consistently across the entire sample population, as well as within subgroups experiencing insufficient sleep (Panel A, Columns (4) and (10) of Table 3). Furthermore, among those experiencing insufficient sleep, better sleep quality correlates with increased levels of risk aversion (Panel A, Column (8) of Table 3). In contrast to previous findings, which report gender differences in the effects of sleep deprivation on risk aversion^26^, we do not observe any gender-based or age-based heterogeneity (Supplementary Table S2).

**Fig. 3:**
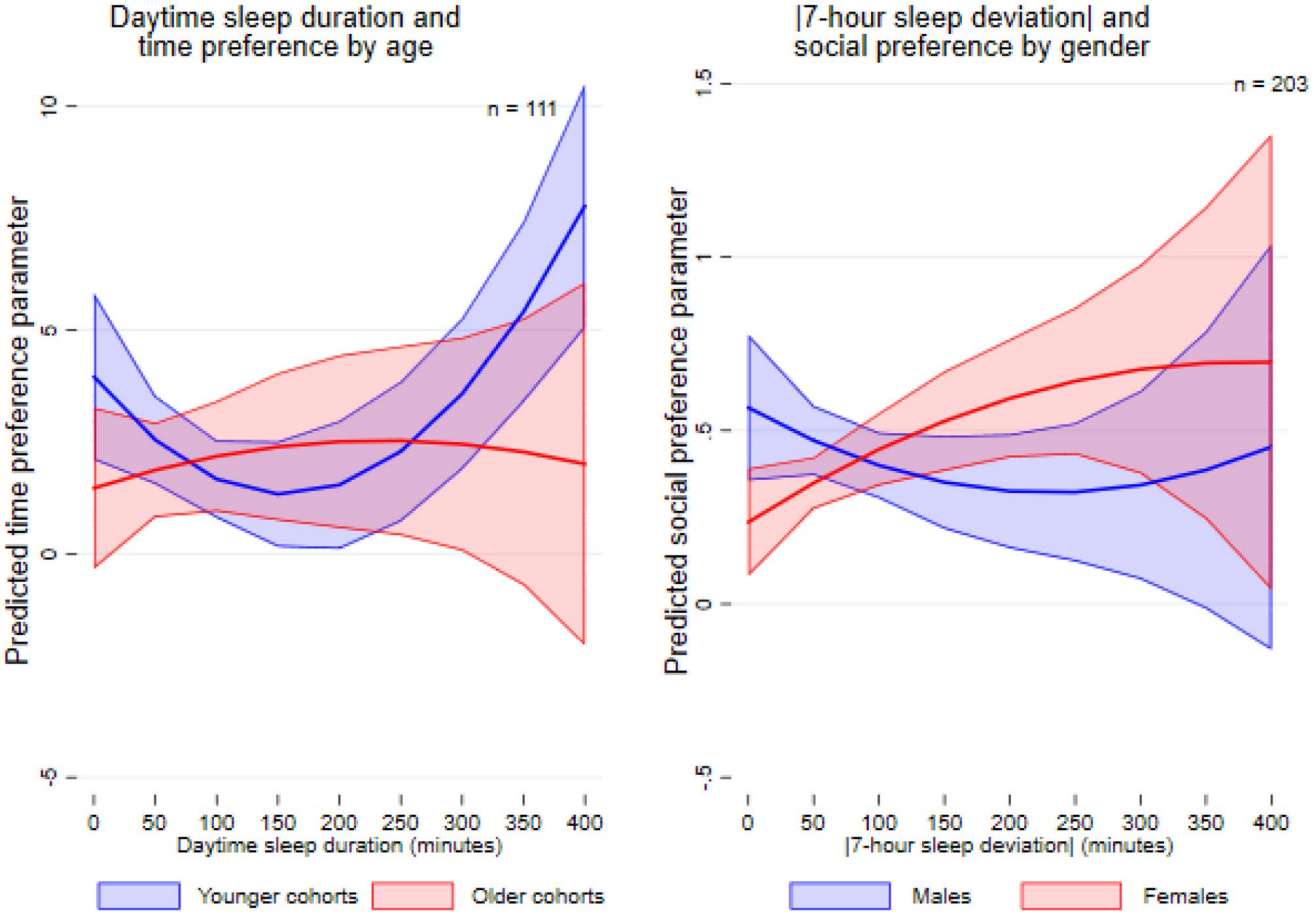
Age-based and gender-based heterogeneity in the relationship between sleep and economic preferences. Notes: Solid lines represent the estimated relationships, while shaded areas indicate 95% confidence intervals. *n* represents the number of individuals.

In terms of time preference, the association with sleep is evident only within the sleep-deprived group. Within this group, longer total daily sleep duration initially correlates with increased impatience, as measured by the IDR. Conversely, for those who take daytime naps, longer daytime sleep duration is associated with a lower IDR, indicating less impatience. These correlations, however, diminish after reaching a certain threshold of sleep duration (Panel B, Columns (4) and (10) of Table 3). We do not find gender-based heterogeneity, but age-based heterogeneity in the relationship between daytime sleep metrics and IDR is evident. For the relatively younger cohorts, defined as those below the median age (48 years) in the sample, taking daytime sleep is negatively correlated with IDR in both the full sample and the sleep-deprived sample, indicating a tendency to be more patient. In contrast, the opposite correlation is found for the relatively older cohorts, whose age is above the median (Columns (5) and (11) of Supplementary Table S3). Additionally, as shown on the left-hand side of Fig. 3, the younger cohorts tend to be more patient when they have longer daytime sleep periods, though this correlation reverses beyond a certain threshold. In contrast, the opposite is observed for the older cohorts (Column (6) of Supplementary Table S3). This statistically significant age-based difference holds only for the full sample.

Regarding social preferences, overall, we do not observe statistically significant correlations between any sleep matrices and aversion to advantageous inequality (Panel C in Table 3). However, gender-based and age-based heterogeneity is found (Supplementary Table S4). As illustrated in the right panel of Fig. 3, for males, there is initially a negative correlation between absolute 7-hour sleep deviation and the guilt parameter, which becomes positive beyond a certain threshold. The opposite pattern is found for females. Among individuals experiencing insufficient sleep, a statistically significant gender difference in the relationship between daytime sleep and social preferences is observed. Males who get daytime sleep tend to be more averse to socially advantageous inequality, whereas females who get daytime sleep tend to be less averse to advantageous inequality. Age-based heterogeneity is documented only within individuals with insufficient sleep. Among them, the relatively younger cohorts with better sleep quality are more averse to advantageous inequality. Initially, longer daytime sleep is correlated with increased aversion to advantageous inequality, but this correlation diminishes and eventually becomes negative as daytime sleep duration increases further. The older cohorts, however, display the opposite pattern.

## Discussion

Despite the importance of sleep, high-quality data on sleep in non-WEIRD populations remain limited. In this study, we collected field data using smartwatches and self-assessments, alongside incentivized economic tasks, to examine sleep patterns and their correlations with individual core economic preferences among smallholder farmers near Bengaluru, India.

Compared to studies on urban poor populations in India, which report shorter, more interrupted sleep^28^, our results suggest that smallholder farmers in India generally experience good sleep, averaging 6.4 hours per night with high sleep quality. This contrast may reflect fewer barriers to sleep, such as noise, light, and crowding, in rural settings. Such heterogeneity within the same country may suggest that sleep behaviors are context-specific, and findings from one population may not generalize to others. Despite this relatively good sleep quality, a substantial portion of our sample (65.2%) still sleeps less than the recommended seven hours per night on average, a common issue associated with reduced attentiveness and impaired decision-making^3,29^. Such insufficient sleep may contribute to persistent productivity challenges in agricultural activities. Additionally, daytime napping is relatively frequent, with 47% of farmers incorporating it into their routines, often as a compensatory response to nighttime sleep deficits.

Consistent with other studies^30^, age and gender statistically significantly influence sleep patterns. Increasing age correlates with shorter total sleep. Women show a distinct sleep duration pattern, sleeping longer than men and more closely aligning with the recommended minimum of seven hours per night. Daytime sleep duration is negatively associated with household size. One plausible explanation is that more people in larger households can create a livelier environment, potentially disrupting the quiet needed for daytime sleep. Additionally, individuals in larger households often have more housework duties, leaving less time available for daytime sleep. As anticipated from literature^31,32^, socioeconomic status positively correlates with sleep quality. However, it is negatively associated with daytime sleep duration, particularly among individuals experiencing insufficient sleep. The substantial correlation size may be attributed to higher social standing being associated with greater social and work responsibilities, increased time demands, and elevated stress and anxiety levels, all of which can lead to shorter and less restful daytime sleep.

While many studies suggest high levels of risk aversion in developing countries^16^, our sample presents a balanced distribution, with 43% risk-averse and 49% as risk-loving participants. Smallholder farmers exhibit high impatience, favoring immediate rewards, similar to findings in other developing countries^33,34^. They show a moderate aversion to advantageous inequality, comparable to levels observed in developed countries^11^. In our sample, sleep correlates with risk and time preferences, but its correlation with social preference is less clear. Nonetheless, sleep correlates with social preference in an age-specific and gender-specific manner. Given that the sampled population exhibits a high level of impatience, a trait also found in other developing country contexts, appropriate sleep interventions addressing sleep deprivation could have far-reaching effects. Beyond direct public health benefits, such interventions may have broader effects on time preferences, potentially leading to better decision-making, enhanced well-being, and improved economic outcomes among the rural poor.

Our results enrich the limited pool of empirical evidence on sleep behavior among smallholder farmers in the rural and semi-rural regions of the Global South. Sleep data obtained from field studies authentically reflects real sleep patterns, enhancing the external validity of research findings. While sleep is a fundamental aspect of human well-being, its role is often overlooked in discussions on poverty and productivity within smallholder farming communities. Our study provides critical datapoints that help identify key issues, laying a valuable foundation for future research. Understanding detailed sleep patterns, sociodemographic correlations, and links to economic preferences not only enriches existing knowledge but also supports the development of targeted interventions addressing both health and economic outcomes in rural areas.

This study opens new avenues for future research. While our data collection was conducted between April and July, which may reflect specific seasonal patterns influenced by temperature and rainfall, expanding data collection across multiple seasons could further enrich the understanding of typical sleep behaviors year-round. Additionally, participants wore smartwatches for an average of four days, which, while capturing valuable short-term insights, limits the ability to analyze variations in sleep behaviors between weekdays and weekends. Future studies with longer observation periods might offer deeper insights while balancing participant compliance. Finally, while our findings highlight statistically significant correlations between sleep metrics and economic preferences, they do not establish causal pathways. Previous research has extensively examined the causal effects of sleep deprivation on risk-taking and social decision-making, with results showing that sleep deprivation promotes increased risk taking and reduced prosociality^2,3,35^. Building on our observations and existing empirical evidence, further investigation into how sleep patterns subsequently affect long-term agricultural productivity and economic outcomes, such as knowledge acquisition, crop management, technology use, or market behaviors, represents a promising area for future research.

## Methods

### Sampling strategy

This study was approved by the Ethics Committee of the University of Göttingen and conducted in accordance with all relevant ethical guidelines and regulations. Participants were informed about the study details and informed consent was obtained from all subjects. Data were collected from smallholder farm households surrounding Bengaluru, the capital city of the southern Indian state of Karnataka, between April and July 2022. To ensure a representative sample, we applied a two-stage stratified sampling strategy. In the first stage, two transects, a northern and a southern one converging at the center of Bengaluru, were identified. Villages along each of these transects were categorized into three strata - rural, semi-rural, and urban - using a Survey Stratification Index^14^. Subsequently, ten villages from each stratum per transect were randomly selected, encompassing approximately one-third of all villages along the transects. In the second stage, an average of 20 households were randomly selected from the chosen villages, with the selection proportional to the village population. While the sampling strategy initially aims for a balanced geographic distribution between urban and rural locations, the data in this study are based solely on smallholder farmers who completed the survey, wore the smartwatch, and participated in the economic tasks, leading to a focus predominantly on rural and semi-rural households.

### Sleeping data

In this study, we collected sleep data using both objective measurements from wrist-worn devices and subjective reports from survey questions. While polysomnography (PSG) is often considered the gold standard for objectively measuring sleep, its invasive nature may disturb individuals’ natural sleep patterns at home, making it impractical for field studies^36^. Recent technological advancements have brought a variety of more cost-effective consumer wearable sleep-trackers, such as wristbands, armbands, smartwatches, and others, which may interfere less with natural sleep patterns. Although the validity of these commercially available wearable devices has been less extensively studied, a review shows that they tend to have a high ability to correctly classify PSG sleep epochs, whether using accelerometry-based or multisensor technology^37^. For this study, the Amazfit Bip U smartwatch was selected based on best-practice recommendations for eliciting sleep data outside of laboratory settings^36^. Each participant wore the same model on their non-dominant hand, where it continuously tracks sleep by monitoring movement through an accelerometer. Data are aggregated with minute-level precision and input into a proprietary algorithm that estimates sleep status. This algorithm further differentiates types of sleep, namely deep or light, and assesses sleep quality, providing a rating that ranges from poor to excellent on a four-point scale. However, research evaluating the accuracy of commercial sleep devices indicates that these technologies typically exhibit lower error and bias when measuring sleep-wake states compared with their performance in quantifying sleep stages such as deep and light sleep^38^. Therefore, this paper focuses exclusively on sleep duration rather than differentiating between deep and light sleep stages. For the self-answered sleep data, participants were asked a series of straightforward recall questions commonly used in field contexts^36^. The questions include: “How many hours per night do you usually sleep?”, “How would you rate the quality of your sleep on a scale from 1-10 (1 represents really bad and 10 is absolutely wonderful)”, “Do you nap during the day?”, and “How often do you nap?”.

Among all respondents who completed the survey, 4% declined to wear the smartwatch, suggesting less concern about self-selection bias in the sample. Participants were free to remove the watches as needed, and no penalties were imposed for doing so. Because revisit dates were not systematically documented, we cannot directly validate the full range of days the watch could have been worn. However, within the observed range, step data, recorded minute by minute and exported at ten-minute intervals, provide supportive evidence that most participants wore the device consistently across consecutive days. Specifically, participants did not exhibit patterns of alternating usage, such as wearing the watch one day, skipping the next, and resuming thereafter. Among the 266 individuals with step data available, only 12 (4.5%) exhibited gaps in step records during their observed wear periods. Excluding these 12 individuals yields results that remain robust to the main findings (Supplementary Tables S5–S6). Additionally, a p-value of 0.167 from the joint F-test confirms no statistically significant difference in sociodemographic characteristics and economic preferences between those who wore the smartwatch longer (above the median of four days) and those who wore it for a shorter duration (Supplementary Table S7).

After removing data inaccuracies caused either by the individual behavior, such as wearing the watch for only certain short periods, or by device errors, the study retains a total of 1,463 observations from 268 individuals. Due to the design structure of the smartwatches, only sleep duration exceeding four hours is assessed for quality, resulting in 994 observations for the sleep quality variable. We categorized observations into nighttime (19:00 - 5:59) and daytime sleep based on local sunrise and sunset times during the months of the study^39–41^. During May, June, and July, when the sleep data were collected, sunrise times ranged between 05:52 and 06:05, and sunset times ranged between 18:34 and 18:50. Therefore, using 19:00 for sunset and 5:59 for sunrise for all participants is justified due to the minimal variation in these months. This classification is based on empirical findings that non-Western communities living closer to natural environments tend to have more stable circadian rhythms, influencing their sleep patterns^42^. Nighttime (daytime) sleep includes any sleep that begins or ends during the night (day), and the total nighttime (daytime) sleep duration is the sum of all sleep periods within the nighttime (daytime). In total, there are 1,229 observations of nighttime sleep from 264 individuals and 234 observations of daytime sleep from 125 individuals.

### Economic preferences data

Individual core economic preferences are elicited through incentivized economic tasks: the EG task for risk preference, the CW task for time preference, and the DG task for social preference. The payoff matrices for each economic task are shown in Supplementary Tables S8–S10.

In the EG task, participants are presented with nine lottery pairs. Each lottery has two possible outcomes, each occurring with a 50% probability. These pairs of lotteries differ in their expected values and variances. Participants are asked to choose one of the nine lotteries they are most likely to play, with their payoff based on the outcome of the selected lottery. A risk-neutral individual would make decisions based purely on expected values, choosing the lottery with the highest expected payoff. Subjects who are risk averse would sacrifice the expected payoff to avoid variance, opting for a safer bet, while risk-seeking subjects would choose a higher-risk option, even if it involves the same or a lower expected payoff^9^. Furthermore, we calculate the CRRA coefficient for each individual to determine the curvature of the individual utility function^15,43^. The risk-neutral choice pattern is located in the CRRA interval (-0.15, 0.15)^15^. A CRRA value above this range reflects a higher level of risk aversion, while a CRRA value below it indicates a more risk-seeking behavior.

In the CW task, participants are asked to choose between two payment options for each of 15 choice alternatives. Option A always offers 120 INR to be received in one week and option B offers 120 + $x$ INR to be received three months and one week later. We applied a delay for the earlier payment to account for quasi-hyperbolic preferences, where individuals strongly favor immediate rewards over future ones. The discount rate is extremely high when choosing between receiving money now versus later, but lower and more stable when the choice is between two future dates^44,45^. The $x$ INR varies across the 15 alternatives, ranging from 110 INR (reflecting a -35% annual interest rate) to 154 INR (reflecting a 100% annual interest rate). When $x$ is zero or negative, we would expect individuals to reject the future option (option B) since it offers no return or even a negative return. As $x$ increases, it is expected that a greater number of participants will choose the future payment option. Consequently, the switching point from option A to option B indicates the range of IDR. However, the underlying assumption is risk neutrality among subjects. Therefore, we correct IDR by accounting for individual utility curvature^45^, i.e., transform the monetary amounts to utilities according to the respective utility function. At the end of the task, one of the 15 alternatives is randomly selected, and participants receive their payment based on their chosen option for that alternative.

In the modified DG, subjects are asked to decide how much of the initial 100 INR they are willing to sacrifice to achieve an equal distribution of payoffs. Specifically, subjects are presented with 11 pairs of payoff vectors and are asked to choose one from each pair. The left payoff vector consistently offers 100 INR for the participant and 0 INR for another person (100, 0), while the right payoff vector offers increasingly equal payoffs, ranging from (0, 0) to (100, 100). The so-called guilt parameter in the Fehr and Schmidt social preference model^46^, i.e., the advantageous inequality aversion, is elicited by determining the egalitarian allocation where the individual is indifferent between keeping the entire endowment (100, 0) and an equal split ($x_i$, $x_i$)^11^.

For the main analysis, we removed subjects who provided incomplete or inconsistent responses in the CW task and the modified DG to help reduce potential noise in the analysis, as done in previous research^11,47,48^. In the CW task, three individuals who did not complete all 15 choices and 22 who switched between two options two or more times are excluded, resulting in a sample size of 243. In the DG task, seven individuals who did not answer all 11 questions and 46 who switched their choices two or more times are excluded, leaving 215 individuals for the analysis.

### Econometric analysis

To explore the relationship between demographic and socioeconomic characteristics and sleep patterns, we applied a mixed-effects regression model. The mix-effects model technique is suitable as it accounts for within-subject correlation by including individual random effects, given the multiple observations of sleep duration for the same individuals across different days. Formally:

$$\begin{aligned} Sleep_{it} = {{\theta }} + \gamma X_{i} + u_i + v_{it} \end{aligned}$$

where $Sleep_{it}$ denotes various sleep matrices for individual *i* on day *t*, $X_{i}$ is a set of observed demographic and socioeconomic characteristics of individual *i*. $u_i$ represents the individual random effect and $v_{it}$ is the error term.

To examine the relationship between sleep and economic preferences, we used Ordinary Least Squares regression with economic preferences as the dependent variables and sleep metrics as explanatory variables, controlling for demographic and socioeconomic factors. Given evidence of a J-shaped relationship between sleep duration and its effects on health, cognitive, economic, and other outcomes^49^, we include a squared term for all sleep duration metrics to capture potential nonlinearity. The model can be described by:

$$\begin{aligned} Preference_{i} = \alpha + \beta _{1} Sleep_{i} + \beta _{2} Sleep_{i}^2 + \delta X_{i} + \epsilon _{i}, \end{aligned}$$

where $Preference_{i}$ represents individual *i*’s risk, time, and social preferences, indicated by CRRA, IDR, and the guilt parameter, respectively. $Sleep_{i}$ denotes various average sleep behaviors of individual *i*, $X_{i}$ is sociodemographic characteristics of individual *i*, and $\epsilon _{i}$ is the error term.

To perform the gender-based and age-based heterogeneity analysis, we interact each sleep metric with a gender dummy or an age dummy. The gender dummy takes the value of 1 for females and 0 for males. The age dummy variable is constructed such that individuals above the median age (older cohorts) are assigned a value of one^28^.

Data processing and analyses were conducted using Stata 15.

## Supplementary Information

Supplementary Information.

## Data Availability

The data and code used in the analysis are available at OSF repository and can be accessed at: https://osf.io/9cg2y/?view_only=91fc4835368548178ee5a0341176bd04.
