# Correction: Sleep patterns, sociodemographic correlates, and their association with economic preferences among Indian smallholder farmers

**DOI:** 10.1038/s41598-025-22557-3

**Published:** 2025-10-16

**Authors:** Hao Luo, Selina Bruns, Oliver Musshoff, Daniel Hermann

**Affiliations:** 1 Department of Agricultural Economics and Rural Development, University of Göttingen, D-37073 Göttingen, Germany; 2 University of Bristol, Bristol, BS8 1QU UK; 3 University of Bonn, D-53115 Bonn, Germany

Correction to: *Scientific reports* 10.1038/s41598-025-06482-z, published online 16 July 2025

The original version of this Article contained errors in Table 3, where the variable names were truncated in the third header row. The correct and incorrect header rows appear below.

Incorrect:


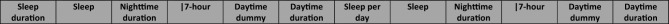


Correct:
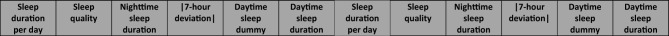


The original Article has been corrected.

