# Supplementary material for: Sleep patterns, sociodemographic correlates, and their association with economic preferences among Indian smallholder farmers
Source: Sci Rep. 2025 Jul 16;15:25782. doi: 10.1038/s41598-025-06482-z (PMC12267576; doi:10.1038/s41598-025-06482-z)
Supplement: Supplementary file 1 — Supplementary Information. [file 41598_2025_6482_MOESM1_ESM.pdf]

**Supplementary Information for**

# Sleep patterns, sociodemographic correlates, and their associations with economic preferences among Indian smallholder farmers

Hao Luo*, [a], Selina Bruns[b], Oliver Musshoff[a], and Daniel Hermann[c]

[a] Department of Agricultural Economics and Rural Development, University of Göttingen, Göttingen D-37073, Germany
[b] Bristol Veterinary School, University of Bristol, Bristol BS8 1QU, UK
[c] Institute for Food and Resource Economics, University of Bonn, Bonn D-53115, Germany

* Corresponding author. Email: hao.luo@uni-goettingen.de

**This PDF file includes:**

Supplementary Notes 1-2
Figure S1
Tables S1 to S10
SI References

**Supplementary Note 1.** Instruction for data collection (English translation).

**A. SURVEY**
We are very happy about your interest and participation!
We are a team of international researchers that wish to understand more about your daily life and your overall living situation. We focus on economic issues and aim to give good policy implications to better support smallholder farmers in Bangaluru, India.

[The consent form is shown to participants, and they agree to it before participating.]

The survey will start with general information about your household, financial activities and perceptions. If applicable, we will finish with some questions about your farm.
We assure you that all information you give during the interview is kept strictly confidential. Data will be used for scientific purposes only and will not be given to any other person. This means, none of your neighbors, your friends, or e.g. the village chief will ever know what you have said during this interview. No one! As a sign of our great appreciation that you take your time for our interview we are happy to compensate you at the end of the session.
We ask you to answer the questions as truthfully as possible – there are not right or wrong answers - as only truthful answers will help us to develop useful recommendations for your village and the region.

This questionnaire is voluntary.

| | |
|---|---|
| Have you understood the instructions and are you willing to participate? | |
| May we come back at a time of your choice? | |
| May we ask for your reasons for refusing? | |
| Do you have any questions that we need to clarify? | |

| HOUSEHOLD SURVEYED: | |
|---|---|
| Transect | |
| Village | |
| Household ID | |
| GPS coordinates of the household | |
| PERSON SURVEYED: | |
| Name of Respondent | |
| Member ID | |
| Are you the household head, i.e. the decision maker when it comes to the farm? | |
| (If no) What is your relation to the household head? | |
| Can you influence decisions related to money and/or farming activities? Please explain: | |
| What is your age in years? | |
| What is your gender? | Female<br>Male |
| What is your marital status? | Married<br>Single<br>Widowed |
| For how many years did you go to school? | |
| What is your religious affiliation? | Hindu<br>Muslim<br>Christian |
| What is your caste? | General<br>Scheduled caste (SC)<br>Scheduled tribe (ST)<br>Other backward class (OBC)<br>Other |
| Roughly speaking, how much income do you have per month? | |
| HOUSEHOLD INFORMATION: | |

| | |
|---|---|
| What is your household size? | |
| How well-off do you consider your household in comparison to other residents of your village? | poorest<br>below average<br>average<br>above average<br>richest |
| SLEEP: | |
| How many hours per night do you usually sleep? | |
| Do you nap during the day? | |
| (If yes) How often do you nap? | |
| How would you rate the average quality of your sleep on a scale from 1-10? | |

**B. SMARTWATCH**
We want to ask you to wear this smartwatch wrist band for the next 5-7 days. The participation in this part of the research is completely voluntary and it is up to you if you want to join or not. This device works and looks just like a watch. It is light and you will not notice it other than you would notice a normal watch. If you agree, the smartwatch will be placed on your non-dominant hand. It is waterproof so you can also wear it while taking a shower or working on the field. It is also heat resistant, so you do not have to worry about it when it is really sunny. We pre-charged it, so you do not have to worry about that either. We would simply ask you to wear it during the next week.
The watch will capture your daily activity (in steps) and you sleep pattern. It will record the hours of sleep and how often you woke up during the night. This information will help us in understanding e.g. your wellbeing and how it is connected to your farm activities. We will only use the data in an aggregated way, i.e. we will not communicate anything about you personally and your personal sleeping pattern to anyone, we will just communicate things like “on average, 600 participants sleep on average 5 hours”.
Do you have any more questions?
Would you voluntarily like to participate in this research and commit to wear the smartwatch wristband for the next 5-7 days? ___________ (Yes/No)

**C. ECONOMIC GAMES**
[The following section applies only when enumerators return to the households to collect the smartwatches.]

We will now start with part 2 of the session and play some games.
You can think of the experiments as games we will play with you. They will be in form of e.g. a quiz, or some lotteries (where you can win actual money)! The survey will cover questions about your business activities and habits, and your personal thoughts.
We are very excited about this session. We will start with two experiments, move on to the survey, then we will have a break where you are invited to relax. Then we will continue with several further experiments and finish with your payout.
Your payout consists of the amount you won in the experiments on top of that! So give it your best.
Please remember that we are not here to judge you. We want to support you, your village and the region. Therefore, all of your truthful information will be of great value to your community. Your answers are completely anonymous!
And always remember: There is no right or wrong answer! There is just your opinion and your answer! And that is the very best.
We are very happy for your help and looking forward to a fun session. If you have any questions, please always ask!

Before we start we would therefore like to ask you how we can transfer the payout to you (easiest way is mobile money). Please state how the money can reach you. Which mobile money service can we use?

**Eckel and Grossman (EG) Task**
We will now play a game. The game is simple. I will show you nine different gambles and you can choose which one you want to play. Once you have made your decision, we will conduct the chosen lottery, and you can win the actual payout. So, we play for real money that you can win on top of your salary for this session!!!

Ok so let's start!
[The payout matrix (Table A1) is shown to the participants.]
As I said, I will show you the different gambles and you tell me which one you want to play. In each gamble, you always have a 50:50 chance to win one amount or the other. In other words, once you decided upon a gamble, I will throw a coin to determine your payout.
So, now let's start the game. I will start with round one and you tell me which option you like best. And remember, you are playing for real money! In round one, you have the 50:50 chance of winning 140 INR or 140 INR. In round 2 (…) Please continue with all rounds and ask at the end: So which round do you choose?
Great job! Now, as promised, you can draw a paper out of this box. There is a number between one and eight and will determine which row we will play for real money.
Finally, please flip the coin for the respective round to determine your payout.

**Coller and Williams (CW) Task**
We will now play one more game. The game is simple. We will play multiple rounds and, in every round, you can choose between 2 Options. Option A and Option B. If you have any questions, please interrupt me anytime. At the end of all rounds we will draw a number and for the respective round, you will get the actual payout! So, we play for real money that you can win on top of your salary for this session!!!
Ok so let's start!
[The payout matrix (Table A2) is shown to the participants.]
As you can see, there are two fields. Field A and Field B. These are the two options you can choose between. Let's start with row one: You can choose between 120 INR in one week – so money you will receive in one week with your payout! – or 110 INR which we will transfer to your bank account in three months and one week. Before you decide, let's take a look at the list. In every row you can get 120 INR in one week. However, the amount you get in three months and one week increases. See for example in row 5, you can choose between 120 INR in one week or 120.90 in three months and one week. In round ten you choose between 120 in one week or 126.15 in three months and one week, and in the last round, you choose between 120 INR in one week or 154 INR in three months and one week. Ok, so you always choose between 120 INR in one week, or a different amount in three months and one week.
Do you have any additional questions?
Okay, then before we start let's make sure you understood the game. Lets say in row 2, can you explain to me what your options are?
Wonderful. Okay, let's start! Row 1: 120 INR in one week or 110 INR in three months and one week, which one do you prefer?
[This process will be repeated for all subsequent rounds.]
Great job! Now, as promised, you can draw a number out of this box. It will be a number between one and 15 and will determine the row which determines your payout.

**Modified Dictator Game (DG)**
We will now play our final game. As in all games before, you again have the chance to win real money which will be added to your overall payout. This time we play a game with two players. Player A (that is you) and Player B (that is someone else). You and I do not know who Player B is, we will anonymously draw that by lot later today.
[The payout matrix (Table A3) is shown to the participants.]
Now, as Player A I will present to you a game with multiple rows and for each row you have to decide between two options. Here is the game (please hand the illustrated version of the game to the participant). I will start explaining the game in row one. Here, you have the choice between option one: Player A gets 100 INR and Player B get's nothing OR you can go for option two, here both players get nothing. Now for the rest of the game, in option 1, Player A will always get 100 INR and Player B will always get 0. What changes is option 2. Let's look at row 2: in option 2 Player A can now get 10 INR and then Player B would also get 10 INR (please continue explaining a few rows by always saying in Option 1 Player A gets 100 INR and Player B gets nothing; in option B both get x).
Please take your time to look at the game and please raise any questions you might have.
Okay, in case you have no more questions, could you just explain to me what options I have in round 1 again? (if it is all correct, you can start the game; otherwise please explain again).
Okay, let's start. Remember that at the end of the game we will determine one row by lot and you can actually win that amount of money. So, for example if you choose option 2 in round 5, Player A will get 40 INR and player B will get 40 INR.
So, round 1, which option do you choose?
[This process will be repeated for all subsequent rounds.]

Great job! Now, as promised, you can draw a number out of this box. It will be a number between one and 11 and will determine the row which determines your payout.
The payment will be determined later once all players played and we randomly and anonymously determined who is Player A and who is PLAYER B.

**Supplementary Note 2.** Agreements between objective and subjective sleep measures.

Fig. S1 presents the comparisons regarding nighttime sleep duration (top left), sleep quality (top right), and whether individuals get daytime sleep (bottom left). Individuals tend to overestimate their nighttime sleep duration by 0.9 hours subjectively, a trend that has also been observed by other studies[1,2]. The correlation between the two measures is statistically significant at the 1% level (p = 0.000), with a correlation coefficient of 0.218 based on Spearman's rank test. The Bland-Altman plot[3] on the top left of Fig. S1 illustrates the agreements between two measures in nighttime sleep duration. The two horizontal green lines are the upper and lower limits of agreement, and the red line in between represents the mean difference between the two measurements across all individuals. The downward-sloping red line indicates that individuals are more likely to overestimate their sleep duration when it is relatively short, and the agreement between self-reported and measured sleep duration increases as their actual sleep duration increases. Similar results are found when comparing self-reports and measured sleep duration by actigraphy[1]. Nonetheless, a few outliers are observed with large discrepancies between self-reported and objective sleep measures. Regarding sleep quality, the correlation between the two measures is very low and not statistically significant (Spearman's correlation coefficient of 0.051, p = 0.408). The top right graphic of Fig. S1 visually shows that there is no clear relationship between self-reported and objectively measured sleep quality. In terms of daytime sleep, the two measures of whether napping occurs are correlated at the 1% level (p = 0.001), with a correlation coefficient of 0.200. The bottom left of Fig. S1 depicts the consistency between the two measures of napping behavior. The red bars, for reference, represent the proportion of self-reported napping behavior in the entire sample, while the blue bars represent the proportion of napping behavior measured by smartwatches in each group, i.e., those who do not report napping and those who report napping. Specifically, the two red bars on the left-hand side indicate those who do not report napping (191 individuals, 71.27%), and the two red bars on the right-hand side indicate those who report napping (77 individuals, 28.73%). For individuals who do not report napping, about 60% of them (114 individuals) do not actually nap, while around 40% of them (77 individuals) do nap based on the smartwatch data. Similarly, for those who report napping, slightly above 60% (48 individuals) take naps, while slightly less than 40% (29 individuals) do not take naps. In summary, approximately 60% of individuals exhibit consistency between their self-reported napping habits and objectively measured napping patterns.

**Fig. S1.** Comparison of self-reported and objectively measured sleep patterns.

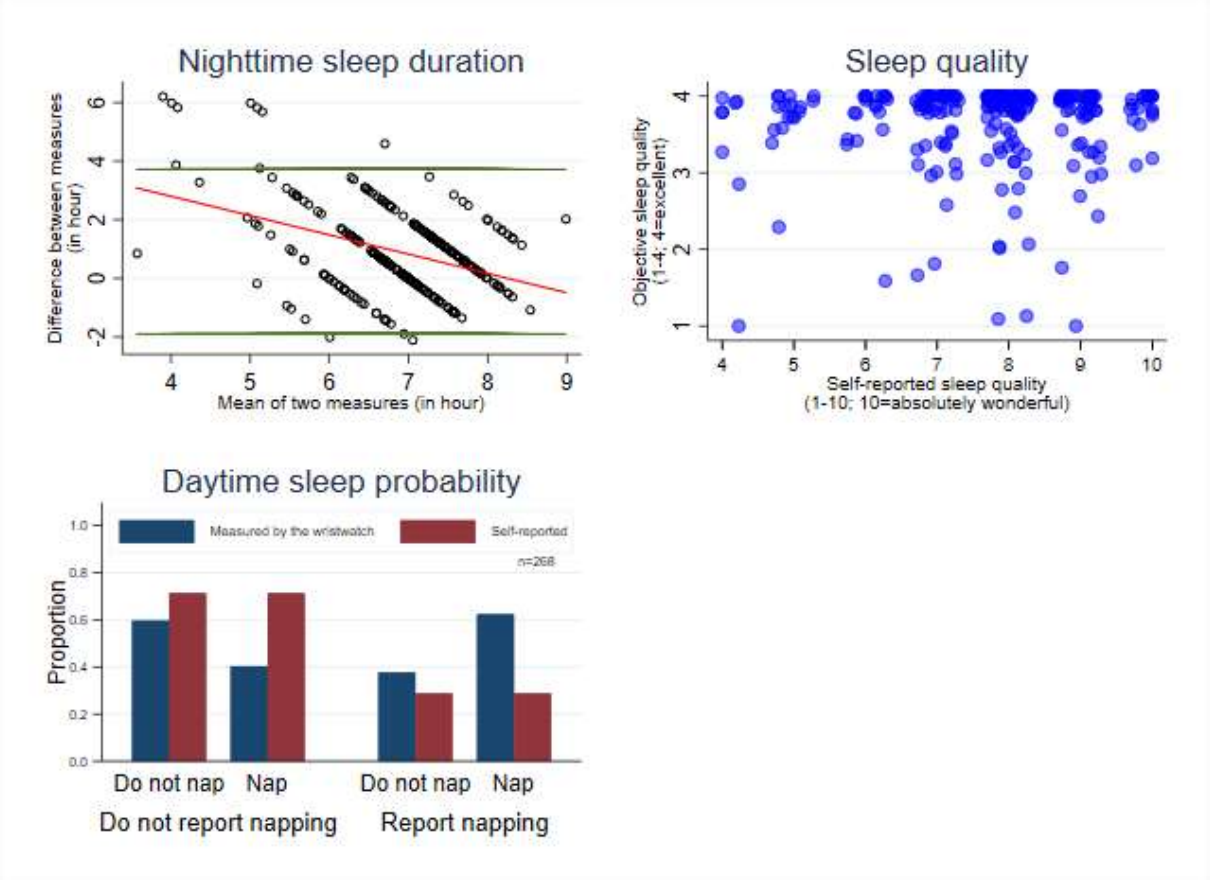


Notes: The top left panel displays a Bland-Altman plot[3] comparing the self-reported and objectively measured nighttime sleep duration. The top right panel illustrates the agreement between self-reported (scale of 1 to 10) and objectively measured (scale of 1 to 4) sleep quality. The bottom panel presents the comparison of self-reported and smartwatch-measured data on whether individuals get daytime sleep. *n* represents the number of individuals.

**Table S1.** Relationship between sleep patterns and individual time and social preferences.

| | Full sample | | | | | | Individuals with insufficient sleep (< 7h) | | | | | |
|---|---|---|---|---|---|---|---|---|---|---|---|---|
| | Sleep duration per day | Sleep quality | Nighttime sleep duration | \|7-hour sleep deviation\| | Daytime sleep dummy | Daytime sleep duration | Sleep duration per day | Sleep quality | Nighttime sleep duration | \|7-hour sleep deviation\| | Daytime sleep dummy | Daytime sleep duration |
| | (1) | (2) | (3) | (4) | (5) | (6) | (7) | (8) | (9) | (10) | (11) | (12) |
| Panel A: Time preference (IDR) | | | | | | | | | | | | |
| X | 0.005 | -0.014 | -0.004 | -0.012* | 0.154 | -0.015 | 0.014** | -0.138 | -0.016 | -0.015* | -0.017 | -0.024** |
| | (0.005) | (0.298) | (0.010) | (0.007) | (0.307) | (0.010) | (0.006) | (0.345) | (0.013) | (0.009) | (0.407) | (0.011) |
| X^2 | 0.000 | | 0.000 | 0.000* | | 0.000* | -0.000** | | 0.000 | 0.000* | | 0.000*** |
| | (0.000) | | (0.000) | (0.000) | | (0.000) | (0.000) | | (0.000) | (0.000) | | (0.000) |
| Constant | 3.870 | 4.075 | 4.865* | 5.036** | 4.568* | 3.628 | 1.336 | 3.973 | 5.665 | 4.808 | 3.702 | 1.101 |
| | (2.864) | (2.533) | (2.797) | (2.298) | (2.370) | (3.441) | (3.412) | (3.437) | (3.765) | (3.160) | (3.225) | (4.463) |
| n | 256 | 249 | 252 | 252 | 256 | 119 | 164 | 159 | 164 | 164 | 164 | 78 |
| Panel B: Social preference (guilt parameter) | | | | | | | | | | | | |
| X | -0.000 | -0.014 | 0.001 | 0.000 | 0.042 | 0.001 | -0.001 | -0.009 | 0.001 | 0.001 | 0.039 | 0.001 |
| | (0.001) | (0.042) | (0.001) | (0.001) | (0.050) | (0.002) | (0.002) | (0.044) | (0.002) | (0.001) | (0.064) | (0.002) |
| X^2 | 0.000 | | 0.000 | -0.000 | | -0.000 | 0.000 | | -0.000 | -0.000 | | -0.000 |
| | (0.000) | | (0.000) | (0.000) | | (0.000) | (0.000) | | (0.000) | (0.000) | | (0.000) |
| Constant | 0.480 | 0.203 | 0.189 | 0.175 | 0.270 | 0.027 | 0.651 | 0.455 | 0.396 | 0.460 | 0.492 | 0.552 |
| | (0.433) | (0.366) | (0.434) | (0.349) | (0.346) | (0.557) | (0.574) | (0.451) | (0.565) | (0.469) | (0.463) | (0.678) |
| n | 251 | 244 | 247 | 247 | 251 | 113 | 161 | 156 | 161 | 161 | 161 | 74 |

Notes: The table presents Ordinary Least Squares regression estimates with individual time and social preferences as the dependent variables and various sleep metrics as the explanatory variables, while controlling for individual demographic and socioeconomic characteristics listed in Panel A of Table 1. The results include both consistent and inconsistent decision-makers. *X^2* reports coefficients on the squared sleep duration, accounting for the nonlinear correlations. Sleep duration is measured in minutes, and sleep quality is rated on a four-point scale, with four indicating the best quality and one the worst. *n* represents the number of individuals. *, **, and *** indicate statistical significance at the 0.10, 0.05, and 0.01 level, respectively. Standard errors are shown in parentheses.

**Table S2. Gender and age heterogeneity in the relationship between sleep patterns and risk preference.**

| | Full sample X = | | | | | | Individuals with insufficient sleep (< 7h) X = | | | | | |
|---|---|---|---|---|---|---|---|---|---|---|---|---|
| | Sleep duration per day (1) | Sleep quality (2) | Nighttime sleep duration (3) | \|7-hour sleep deviation\| (4) | Daytime sleep dummy (5) | Daytime sleep duration (6) | Sleep duration per day (7) | Sleep quality (8) | Nighttime sleep duration (9) | \|7-hour sleep deviation\| (10) | Daytime sleep dummy (11) | Daytime sleep duration (12) |
| X | 0.000 | 0.095 | -0.000 | 0.007** | 0.058 | 0.005 | -0.002 | 0.163 | 0.006 | 0.007** | 0.035 | 0.008 |
| | (0.003) | (0.115) | (0.004) | (0.003) | (0.133) | (0.006) | (0.004) | (0.131) | (0.005) | (0.003) | (0.175) | (0.007) |
| X^2 | 0.000 | | -0.000 | -0.000** | | -0.000 | 0.000 | | -0.000 | -0.000* | | -0.000 |
| | (0.000) | | (0.000) | (0.000) | | (0.000) | (0.000) | | (0.000) | (0.000) | | (0.000) |
| X * Female | 0.000 | -0.001 | -0.002 | -0.004 | -0.174 | -0.005 | 0.001 | -0.047 | -0.006 | -0.003 | -0.228 | -0.007 |
| | (0.004) | (0.146) | (0.006) | (0.004) | (0.181) | (0.007) | (0.005) | (0.166) | (0.008) | (0.005) | (0.223) | (0.008) |
| X^2 * Female | 0.000 | | 0.000 | 0.000 | | 0.000 | 0.000 | | 0.000 | 0.000 | | 0.000 |
| | (0.000) | | (0.000) | (0.000) | | (0.000) | (0.000) | | (0.000) | (0.000) | | (0.000) |
| Constant | -0.959 | -1.088 | -0.763 | -1.208* | -0.874 | -0.261 | 0.243 | -0.763 | -0.818 | -0.757 | -0.241 | -0.386 |
| | (0.872) | (0.712) | (0.899) | (0.639) | (0.598) | (1.259) | (1.044) | (0.793) | (1.108) | (0.801) | (0.750) | (1.590) |
| n | 258 | 251 | 254 | 254 | 258 | 119 | 166 | 161 | 166 | 166 | 166 | 78 |
| | | | | | | | | | | | | |
| X | 0.001 | 0.117 | 0.000 | 0.005** | -0.012 | 0.004 | 0.000 | 0.157 | 0.004 | 0.005 | -0.063 | 0.006 |
| | (0.003) | (0.110) | (0.004) | (0.003) | (0.119) | (0.004) | (0.004) | (0.133) | (0.005) | (0.003) | (0.154) | (0.005) |
| X^2 | -0.000 | | -0.000 | -0.000* | | -0.000* | -0.000 | | -0.000 | -0.000 | | -0.000* |
| | (0.000) | | (0.000) | (0.000) | | (0.000) | (0.000) | | (0.000) | (0.000) | | (0.000) |
| X * Median age | -0.001 | -0.025 | -0.004 | -0.001 | -0.051 | -0.009 | -0.006 | -0.028 | -0.002 | 0.001 | -0.045 | -0.006 |
| | (0.004) | (0.143) | (0.008) | (0.004) | (0.177) | (0.006) | (0.007) | (0.168) | (0.009) | (0.005) | (0.236) | (0.008) |
| X^2 * Median age | 0.000 | | 0.000 | 0.000 | | 0.000* | 0.000 | | 0.000 | -0.000 | | 0.000 |
| | (0.000) | | (0.000) | (0.000) | | (0.000) | (0.000) | | (0.000) | (0.000) | | (0.000) |
| Constant | -0.815 | -1.002 | -0.600 | -0.929 | -0.634 | 0.235 | -0.183 | -0.777 | -0.485 | -0.508 | -0.078 | -0.120 |
| | (0.876) | (0.735) | (0.901) | (0.635) | (0.620) | (1.141) | (1.173) | (0.839) | (1.086) | (0.766) | (0.756) | (1.395) |
| n | 258 | 251 | 254 | 254 | 258 | 119 | 166 | 161 | 166 | 166 | 166 | 78 |

Notes: The table presents regression estimates with risk preference (CRRA) as the dependent variables and various sleep metrics as the explanatory variables, considering gender-based and age-based heterogeneity. All specifications control for individual sociodemographic characteristics listed in Panel A of Table 1. *X^2* reports coefficients on the squared sleep duration, accounting for nonlinear relationship. Both *X* and *X^2* are interacted with dummies indicating whether the individual is female (*Female*) or above the median age (*Median age*). Sleep duration is measured in minutes, and sleep quality is rated on a four-point scale, with four indicating the best quality and one the worst. *n* represents the number of individuals. *, **, and *** indicate statistical significance at the 0.10, 0.05, and 0.01 level, respectively. Standard errors are shown in parentheses.

**Table S3.** Gender and age heterogeneity in the relationship between sleep patterns and time preference.

| | Full sample X = | | | | | | Individuals with insufficient sleep (< 7h) X = | | | | | |
|---|---|---|---|---|---|---|---|---|---|---|---|---|
| | Sleep duration per day (1) | Sleep quality (2) | Nighttime sleep duration (3) | \|7-hour sleep deviation\| (4) | Daytime sleep dummy (5) | Daytime sleep duration (6) | Sleep duration per day (7) | Sleep quality (8) | Nighttime sleep duration (9) | \|7-hour sleep deviation\| (10) | Daytime sleep dummy (11) | Daytime sleep duration (12) |
| X | 0.004 | -0.269 | 0.002 | -0.018 | -0.406 | -0.035** | 0.018* | -0.287 | -0.015 | -0.02 | -0.784 | -0.043** |
| | (0.012) | (0.496) | (0.014) | (0.011) | (0.461) | (0.016) | (0.010) | (0.549) | (0.019) | (0.014) | (0.615) | (0.020) |
| X^2 | -0.000 | | -0.000 | 0.000 | | 0.000** | -0.000 | | 0.000 | 0.000 | | 0.000** |
| | (0.000) | | (0.000) | (0.000) | | (0.000) | (0.000) | | (0.000) | (0.000) | | (0.000) |
| X * Female | 0.004 | 0.375 | -0.013 | 0.015 | 0.969 | 0.025 | -0.006 | 0.197 | -0.003 | 0.01 | 1.278 | 0.03 |
| | (0.013) | (0.679) | (0.019) | (0.014) | (0.630) | (0.021) | (0.014) | (0.765) | (0.027) | (0.019) | (0.815) | (0.026) |
| X^2 * Female | -0.000 | | 0.000 | -0.000 | | -0.000 | 0.000 | | -0.000 | -0.000 | | -0.000 |
| | (0.000) | | (0.000) | (0.000) | | (0.000) | (0.000) | | (0.000) | (0.000) | | (0.000) |
| Constant | 3.486 | 4.245 | 3.076 | 5.104** | 4.334* | 4.908 | 0.437 | 3.901 | 4.544 | 5.078 | 4.242 | 4.357 |
| | (3.391) | (2.907) | (3.150) | (2.419) | (2.364) | (3.734) | (3.489) | (3.727) | (4.333) | (3.511) | (3.211) | (5.099) |
| n | 234 | 227 | 230 | 230 | 234 | 111 | 148 | 143 | 148 | 148 | 148 | 72 |
| | | | | | | | | | | | | |
| X | 0.002 | 0.05 | -0.007 | -0.011 | -0.446 | -0.034*** | 0.012 | -0.018 | -0.015 | -0.007 | -0.76 | -0.039** |
| | (0.009) | (0.548) | (0.012) | (0.011) | (0.445) | (0.013) | (0.009) | (0.651) | (0.021) | (0.014) | (0.574) | (0.015) |
| X^2 | -0.000 | | 0.000 | 0.000 | | 0.000*** | -0.000 | | 0.000 | 0.000 | | 0.000*** |
| | (0.000) | | (0.000) | (0.000) | | (0.000) | (0.000) | | (0.000) | (0.000) | | (0.000) |
| X * Median age | 0.007 | -0.22 | 0.007 | 0.001 | 1.175* | 0.043** | 0.022 | -0.265 | -0.005 | -0.015 | 1.476* | 0.035 |
| | (0.012) | (0.674) | (0.021) | (0.015) | (0.618) | (0.019) | (0.021) | (0.783) | (0.029) | (0.019) | (0.845) | (0.030) |
| X^2 * Median age | -0.000 | | -0.000 | 0.000 | | -0.000*** | -0.000 | | 0.000 | 0.000 | | -0.000 |
| | (0.000) | | (0.000) | (0.000) | | (0.000) | (0.000) | | (0.000) | (0.000) | | (0.000) |
| Constant | 2.981 | 2.753 | 3.374 | 4.209* | 4.113* | 3.927 | 2.172 | 2.995 | 4.729 | 4.192 | 3.763 | 5.026 |
| | (3.166) | (3.084) | (3.293) | (2.512) | (2.395) | (3.509) | (3.761) | (4.038) | (4.518) | (3.530) | (3.277) | (4.887) |
| n | 234 | 227 | 230 | 230 | 234 | 111 | 148 | 143 | 148 | 148 | 148 | 72 |

Notes: The table presents regression estimates with time preference (IDR) as the dependent variables and various sleep metrics as the explanatory variables, considering gender-based and age-based heterogeneity. All specifications control for individual sociodemographic characteristics listed in Panel A of Table 1. *X^2* reports coefficients on the squared sleep duration, accounting for nonlinear relationship. Both *X* and *X^2* are interacted with dummies indicating whether the individual is female (*Female*) or above the median age (*Median age*). Sleep duration is measured in minutes, and sleep quality is rated on a four-point scale, with four indicating the best quality and one the worst. *n* represents the number of individuals. *, **, and *** indicate statistical significance at the 0.10, 0.05, and 0.01 level, respectively. Standard errors are shown in parentheses.

**Table S4.** Gender and age heterogeneity in the relationship between sleep patterns and social preference.

| | Full sample X = | | | | | | Individuals with insufficient sleep (< 7h) X = | | | | | |
|---|---|---|---|---|---|---|---|---|---|---|---|---|
| | Sleep duration per day | Sleep quality | Nighttime sleep duration | \|7-hour sleep deviation\| | Daytime sleep dummy | Daytime sleep duration | Sleep duration per day | Sleep quality | Nighttime sleep duration | \|7-hour sleep deviation\| | Daytime sleep dummy | Daytime sleep duration |
| | (1) | (2) | (3) | (4) | (5) | (6) | (7) | (8) | (9) | (10) | (11) | (12) |
| X | -0.002 | 0.011 | 0.000 | -0.002 | -0.019 | 0.005* | -0.000 | 0.019 | -0.002 | -0.002 | -0.136 | 0.004 |
| | (0.002) | (0.060) | (0.002) | (0.002) | (0.081) | (0.003) | (0.002) | (0.063) | (0.003) | (0.002) | (0.100) | (0.003) |
| X^2 | 0.000 | | 0.000 | 0.000 | | -0.000** | 0.000 | | 0.000 | 0.000 | | -0.000* |
| | (0.000) | | (0.000) | (0.000) | | (0.000) | (0.000) | | (0.000) | (0.000) | | (0.000) |
| X * Female | 0.002 | -0.057 | -0.003 | 0.005** | 0.124 | -0.004 | 0.001 | -0.031 | 0.004 | 0.005* | 0.287** | -0.005 |
| | (0.002) | (0.102) | (0.003) | (0.002) | (0.109) | (0.004) | (0.004) | (0.106) | (0.004) | (0.003) | (0.135) | (0.005) |
| X^2 * Female | -0.000 | | 0.000 | -0.000 | | 0.000* | -0.000 | | -0.000* | -0.000 | | 0.000* |
| | (0.000) | | (0.000) | (0.000) | | (0.000) | (0.000) | | (0.000) | (0.000) | | (0.000) |
| Constant | 0.656 | 0.162 | 0.158 | 0.456 | 0.360 | -0.34 | 0.918 | 0.714 | 0.991 | 1.098 | 1.135** | -0.112 |
| | (0.521) | (0.434) | (0.546) | (0.430) | (0.411) | (0.599) | (0.750) | (0.565) | (0.712) | (0.595) | (0.553) | (0.764) |
| n | 206 | 201 | 203 | 203 | 206 | 92 | 133 | 129 | 133 | 133 | 133 | 60 |
| X | -0.002 | -0.117 | -0.001 | -0.000 | 0.074 | -0.001 | 0.000 | -0.145 | -0.000 | -0.000 | 0.060 | -0.003 |
| | (0.002) | (0.095) | (0.002) | (0.002) | (0.075) | (0.003) | (0.002) | (0.098) | (0.003) | (0.002) | (0.094) | (0.004) |
| X^2 | 0.000 | | 0.000 | 0.000 | | 0.000 | -0.000 | | -0.000 | 0.000 | | 0.000 |
| | (0.000) | | (0.000) | (0.000) | | (0.000) | (0.000) | | (0.000) | (0.000) | | (0.000) |
| X * Median age | 0.003 | 0.145 | 0.002 | 0.001 | -0.062 | 0.005 | 0.000 | 0.194* | 0.002 | 0.002 | -0.114 | 0.009* |
| | (0.002) | (0.108) | (0.003) | (0.002) | (0.112) | (0.003) | (0.004) | (0.109) | (0.004) | (0.003) | (0.145) | (0.004) |
| X^2 * Median age | -0.000 | | -0.000 | -0.000 | | -0.000 | 0.000 | | -0.000 | -0.000 | | -0.000*** |
| | (0.000) | | (0.000) | (0.000) | | (0.000) | (0.000) | | (0.000) | (0.000) | | (0.000) |
| Constant | 0.746 | 0.715 | 0.535 | 0.322 | 0.337 | 0.287 | 0.963 | 1.230* | 1.025 | 0.876 | 0.898 | 0.488 |
| | (0.542) | (0.540) | (0.557) | (0.443) | (0.431) | (0.641) | (0.761) | (0.621) | (0.706) | (0.630) | (0.600) | (0.761) |
| n | 206 | 201 | 203 | 203 | 206 | 92 | 133 | 129 | 133 | 133 | 133 | 60 |

Notes: The table presents regression estimates with social preference (guilt parameter) as the dependent variables and various sleep metrics as the explanatory variables, considering gender-based and age-based heterogeneity. All specifications control for individual sociodemographic characteristics listed in Panel A of Table 1. *X^2* reports coefficients on the squared sleep duration, accounting for nonlinear relationship. Both *X* and *X^2* are interacted with dummies indicating whether the individual is female (*Female*) or above the median age (*Median age*). Sleep duration is measured in minutes, and sleep quality is rated on a four-point scale, with four indicating the best quality and one the worst. *n* represents the number of individuals. *, **, and *** indicate statistical significance at the 0.10, 0.05, and 0.01 level, respectively. Standard errors are shown in parentheses.

**Table S5.** Relationship between demographic and socioeconomic characteristics and sleep patterns.

| | Full sample | | | | | Individuals with insufficient sleep (< 7h) | | | | |
|---|---|---|---|---|---|---|---|---|---|---|
| | Sleep duration per day | Sleep quality | Nighttime sleep duration | \|7-hour sleep deviation\| | Daytime sleep duration | Sleep duration per day | Sleep quality | Nighttime sleep duration | \|7-hour sleep deviation\| | Daytime sleep duration |
| | (1) | (2) | (3) | (4) | (5) | (6) | (7) | (8) | (9) | (10) |
| Age (in years) | -6.512** | -0.002 | -5.502** | -1.172 | -1.081 | -0.799 | 0.035 | -0.003 | -2.018 | -6.886 |
| | (2.627) | (0.017) | (2.412) | (1.488) | (3.664) | (2.995) | (0.036) | (2.803) | (2.735) | (6.391) |
| Age^2 (in years) | 0.061** | 0.000 | 0.050** | 0.013 | 0.005 | 0.002 | -0.000 | -0.008 | 0.026 | 0.064 |
| | (0.025) | (0.000) | (0.023) | (0.014) | (0.033) | (0.029) | (0.000) | (0.026) | (0.026) | (0.058) |
| Female | 19.320* | 0.100 | 16.582 | -15.37** | 9.355 | 15.472 | 0.058 | 17.545 | -24.914** | 3.596 |
| | (11.544) | (0.082) | (10.267) | (7.433) | (14.045) | (11.580) | (0.116) | (11.025) | (10.162) | (18.181) |
| Married | 7.509 | 0.0316 | -0.424 | -0.314 | -1.603 | 3.531 | 0.119 | 3.601 | -3.998 | -11.430 |
| | (16.612) | (0.100) | (13.935) | (9.727) | (18.294) | (18.52) | (0.145) | (16.271) | (14.547) | (20.998) |
| Religion (Hindu) | 12.299 | -0.007 | -26.431 | 17.749 | -7.945 | -47.705** | 0.196 | -17.505 | 37.318** | 34.484* |
| | (59.885) | (0.152) | (16.325) | (14.380) | (19.710) | (21.815) | (0.254) | (21.063) | (18.737) | (20.833) |
| Caste (General) | 4.319 | -0.042 | -1.416 | -5.150 | -4.493 | 9.419 | 0.026 | 7.069 | -10.665 | 13.701 |
| | (10.673) | (0.072) | (9.493) | (6.989) | (15.928) | (11.600) | (0.101) | (11.019) | (10.305) | (17.131) |
| Household size | 0.765 | -0.000 | -0.294 | -0.646 | -2.341 | 1.912 | -0.000 | 0.550 | -0.993 | -3.802* |
| | (1.694) | (0.009) | (1.549) | (1.038) | (1.482) | (1.364) | (0.011) | (1.275) | (1.137) | (1.958) |
| Education (in years) | 0.302 | -0.003 | -0.027 | -0.606 | -0.525 | -0.326 | -0.017 | -0.844 | -0.450 | 0.307 |
| | (1.494) | (0.010) | (1.347) | (0.957) | (1.875) | (1.548) | (0.013) | (1.369) | (1.248) | (1.679) |
| Monthly income | -0.023 | 0.003 | -0.170 | -0.036 | 0.462 | 0.296 | 0.002 | -0.182 | 0.071 | 1.440* |
| (in 1,000 INR) | (0.409) | (0.002) | (0.455) | (0.342) | (0.823) | (0.468) | (0.004) | (0.491) | (0.455) | (0.852) |
| Social status | -0.349 | 0.171* | -9.823 | 12.070 | -0.626 | 0.295 | 0.335** | 1.608 | 7.540 | -41.181** |
| | (14.144) | (0.096) | (13.422) | (10.360) | (14.235) | (17.150) | (0.169) | (17.593) | (16.160) | (17.508) |
| Living area | 1.728 | -0.012 | -6.347 | 7.315 | 11.006 | 4.611 | 0.022 | -10.041 | 7.508 | 27.507** |
| | (11.763) | (0.075) | (10.640) | (7.748) | (12.540) | (12.047) | (0.105) | (11.721) | (10.650) | (13.378) |
| Constant | 505.842*** | 3.409*** | 586.274*** | 58.264 | 103.972 | 391.489*** | 1.922* | 403.472*** | 77.701 | 221.143 |
| | (98.377) | (0.573) | (76.680) | (51.033) | (90.616) | (96.331) | (1.051) | (88.041) | (82.172) | (149.834) |
| N | 1,076 | 923 | 1,025 | 1,025 | 189 | 695 | 563 | 664 | 664 | 120 |

Notes: The table presents the results from the mixed-effects regression analysis, including individual random effects. Individuals with step-level interruptions during the observed wear period are excluded from these results. *N* represents the number of observations. Each observation in the regression represents the total sleep aggregated for either the entire day, nighttime, or daytime. Sleep duration is measured in minutes, and sleep quality is rated on a four-point scale, with four indicating the best quality and one the worst. *Social status comparison* is a self-assessment of household social standing on a scale from 1 (low), 2 (average), to 3 (high). *Living area* is categorized from 1 (rural), 2 (semi-rural), to 3 (urban). Column (5) includes only individuals who get daytime sleep and Column (10) includes those who get daytime sleep within the subgroup experiencing insufficient nighttime sleep. *, **, and *** indicate statistical significance at the 0.10, 0.05, and 0.01 level, respectively. Standard errors are shown in parentheses.

**Table S6.** Relationship between sleep patterns and individual economic preferences.

| | Full sample | | | | | | Individuals with insufficient sleep (< 7h) | | | | | |
|---|---|---|---|---|---|---|---|---|---|---|---|---|
| | Sleep duration per day (1) | Sleep quality (2) | Nighttime sleep duration (3) | \|7-hour sleep deviation\| (4) | Daytime sleep dummy (5) | Daytime sleep duration (6) | Sleep duration per day (7) | Sleep quality (8) | Nighttime sleep duration (9) | \|7-hour sleep deviation\| (10) | Daytime sleep dummy (11) | Daytime sleep duration (12) |
| Panel A: Risk preference (CRRA) | | | | | | | | | | | | |
| X | -0.002 | 0.061 | -0.001 | 0.004* | -0.035 | 0.001 | -0.003 | 0.100 | 0.002 | 0.004* | -0.079 | 0.004 |
| | (0.003) | (0.074) | (0.003) | (0.002) | (0.095) | (0.003) | (0.004) | (0.087) | (0.004) | (0.002) | (0.120) | (0.003) |
| X^2 | 0.000 | | 0.000 | -0.000* | | -0.000 | 0.000 | | -0.000 | -0.000 | | -0.000* |
| | (0.000) | | (0.000) | (0.000) | | (0.000) | (0.000) | | (0.000) | (0.000) | | (0.000) |
| Constant | -0.426 | -0.557 | 0.309 | 0.582 | -0.389 | 0.210 | 0.212 | -0.471 | -0.226 | 0.368 | 0.088 | -0.041 |
| | (0.842) | (0.704) | (0.825) | (0.646) | (0.641) | (1.133) | (1.067) | (0.826) | (1.005) | (0.0792) | (0.795) | (1.328) |
| n | 245 | 238 | 241 | 241 | 245 | 112 | 159 | 154 | 159 | 159 | 159 | 74 |
| Panel B: Time preference (IDR) | | | | | | | | | | | | |
| X | 0.008 | 0.088 | -0.003 | -0.010 | 0.094 | -0.018* | 0.018** | 0.029 | -0.015 | -0.013 | -0.189 | -0.025** |
| | (0.008) | (0.331) | (0.010) | (0.007) | (0.333) | (0.010) | (0.008) | (0.386) | (0.014) | (0.010) | (0.443) | (0.011) |
| X^2 | -0.000 | | 0.000 | 0.000 | | 0.000* | -0.000** | | 0.000 | 0.000 | | 0.000*** |
| | (0.000) | | (0.000) | (0.000) | | (0.000) | (0.000) | | (0.000) | (0.000) | | (0.000) |
| Constant | 2.103 | 1.848 | 2.914 | 3.291 | 2.866 | 3.585 | 1.386 | 3.102 | 5.149 | 4.677 | 3.685 | 2.815 |
| | (3.125) | (2.874) | (2.977) | (2.551) | (2.573) | (3.584) | (3.674) | (3.820) | (4.004) | (3.513) | (3.486) | (4.757) |
| n | 223 | 216 | 219 | 219 | 223 | 106 | 142 | 137 | 142 | 142 | 142 | 69 |
| Panel B: Social preference (guilt parameter) | | | | | | | | | | | | |
| X | -0.002 | -0.041 | -0.000 | 0.000 | 0.054 | 0.002 | 0.001 | -0.031 | 0.000 | 0.001 | 0.022 | 0.000 |
| | (0.002) | (0.052) | (0.002) | (0.001) | (0.057) | (0.002) | (0.002) | (0.056) | (0.002) | (0.001) | (0.069) | (0.003) |
| X^2 | 0.000 | | 0.000 | -0.000 | | -0.000 | -0.000 | | -0.000 | -0.000 | | 0.000 |
| | (0.000) | | (0.000) | (0.000) | | (0.000) | (0.000) | | (0.000) | (0.000) | | (0.000) |
| Constant | 0.382 | 0.084 | 0.136 | 0.009 | 0.079 | -0.054 | 0.680 | 0.635 | 0.741 | 0.657 | 0.727 | 0.490 |
| | (0.489) | (0.438) | (0.507) | (0.421) | (0.406) | (0.575) | (0.667) | (0.548) | (0.673) | (0.565) | (0.548) | (0.660) |
| n | 194 | 189 | 191 | 191 | 194 | 85 | 126 | 122 | 126 | 126 | 126 | 56 |

Notes: The table presents Ordinary Least Squares regression estimates with individual time and social preferences as the dependent variables and various sleep metrics as the explanatory variables, while controlling for individual demographic and socioeconomic characteristics listed in Panel A of Table 1. Individuals with step-level interruptions during the observed wear period are excluded from these results. The results include both consistent and inconsistent decision-makers. *X^2* reports coefficients on the squared sleep duration, accounting for the nonlinear correlations. Sleep duration is measured in minutes, and sleep quality is rated on a four-point scale, with four indicating the best quality and one the worst. *n* represents the number of individuals. *, **, and *** indicate statistical significance at the 0.10, 0.05, and 0.01 level, respectively. Standard errors are shown in parentheses.

**Table S7.** Comparison of sociodemographic characteristics, self-assessed sleep patterns and economic preferences between participants wearing the smartwatch below vs. above the median day (4 days)

| | **Below the median wear-day** | | | **Above the median wear-day** | | | **Difference** |
|---|---|---|---|---|---|---|---|
| | Mean | SD | n | Mean | SD | n | |
| Age (in years) | 47.605 | 13.335 | 147 | 48.884 | 13.918 | 121 | 1.279 |
| Female | 0.531 | - | 147 | 0.504 | - | 121 | -0.027 |
| Married | 0.857 | - | 147 | 0.843 | - | 121 | 0.006 |
| Religion (Hindu) | 0.972 | - | 145 | 0.983 | - | 121 | 0.011 |
| Caste (General) | 0.507 | - | 146 | 0.537 | - | 121 | 0.030 |
| Household size | 4.597 | 2.097 | 144 | 5.067 | 2.987 | 120 | -0.043 |
| Education (in years) | 6.272 | 4.980 | 147 | 6.496 | 4.659 | 121 | 0.224 |
| Monthly income (in 1,000 INR) | 10.992 | 11.216 | 144 | 12.888 | 11.151 | 121 | 1.016 |
| Social status comparison | 2 | 0.468 | 147 | 2.041 | 0.396 | 121 | 0.041 |
| Living area | 2.733 | 0.474 | 146 | 2.683 | 0.467 | 120 | -0.054 |
| Sleep duration per night (in minutes) | 437.143 | 55.295 | 147 | 441.322 | 51.299 | 121 | 4.599 |
| Sleep quality (1-10 scale) | 7.728 | 1.393 | 147 | 7.628 | 1.403 | 121 | -0.100 |
| Nap (yes = 1; no = 0) | 0.333 | - | 147 | 0.231 | - | 121 | -0.012* |
| Risk preference: CRRA | -0.088 | 0.672 | 147 | -0.121 | 0.716 | 121 | -0.033 |
| Time preference: IDR | 1.967 | 2.269 | 145 | 2.040 | 2.318 | 120 | 0.073 |
| Social preference: Guilt parameter | 0.479 | 0.381 | 145 | 0.456 | 0.393 | 116 | -0.023 |
| Joint test (F-test [p-value]) | | | | | | | 1.350<br>[0.167] |

Notes: *n* represents the number of individuals. The column *Difference* displays mean differences in sociodemographic characteristics and economic preferences between individuals who wore the smartwatch for fewer versus more than the median of four days. The Mann-Whitney U test is used for continuous variables, and the Chi-squared test is applied for categorical variables. *, **, and *** indicate statistical significance at the 0.10, 0.05, and 0.01 level, respectively.

**Table S8.** Payoff matrix for the measurement of risk preference.

| | **Expected payoff (INR)** | **Probability (%)** | **Payout A (INR)** | **Probability (%)** | **Payout B (INR)** | **Risk preference classification** | **Range of RRA for $U(x) = \frac{x^{1-r}}{1-r}$** |
|---|---|---|---|---|---|---|---|
| 1 | 140 | 50 | 140 | 50 | 140 | Risk averse | r > 1.37 |
| 2 | 145 | 50 | 110 | 50 | 180 | | 0.97 < r < 1.37 |
| 3 | 157.5 | 50 | 85 | 50 | 230 | | 0.68 < r < 0.97 |
| 4 | 172.5 | 50 | 65 | 50 | 280 | | 0.41 < r < 0.68 |
| 5 | 177.5 | 50 | 50 | 50 | 305 | | 0.15 < r < 0.41 |
| 6 | 181.5 | 50 | 40 | 50 | 323 | Risk neutral | -0.15 < r < 0.15 |
| 7 | 181 | 50 | 35 | 50 | 327 | Risk seeking | -0.49 < r < -0.15 |
| 8 | 179 | 50 | 30 | 50 | 328 | | -0.95 < r < -0.49 |
| 9 | 172.5 | 50 | 15 | 50 | 330 | | r < -0.95 |

Notes: The last two columns were not shown to the participants. Individuals are asked to choose one of the nine payoff alternatives that they prefer most for receiving payment.

**Table S9.** Payoff matrix for the measurement of time preference.

| | **Option A (INR) in one week** | **Option B (INR) in 3 months + one week** | **Annual interest rate (%)** | **Annual effective interest rate (%)** | **Preferred payment option (A or B)** |
|---|---|---|---|---|---|
| 1 | 120 | 110 | -35 | -29.74 | A O B |
| 2 | 120 | 120 | 0 | 0 | A O B |
| 3 | 120 | 120.3 | 1 | 1 | A O B |
| 4 | 120 | 120.6 | 2 | 2.02 | A O B |
| 5 | 120 | 120.9 | 3 | 3.04 | A O B |
| 6 | 120 | 121.3 | 4 | 4.4 | A O B |
| 7 | 120 | 121.5 | 5 | 5.08 | A O B |
| 8 | 120 | 123 | 10 | 10.34 | A O B |
| 9 | 120 | 124.6 | 15 | 16.13 | A O B |
| 10 | 120 | 126.15 | 20 | 21.94 | A O B |
| 11 | 120 | 127.5 | 24 | 27.15 | A O B |
| 12 | 120 | 129.4 | 30 | 34.72 | A O B |
| 13 | 120 | 136 | 50 | 63.37 | A O B |
| 14 | 120 | 145 | 76 | 108.5 | A O B |
| 15 | 120 | 154 | 100 | 161.11 | A O B |

Notes: The annual effective interest rate is calculated based on the daily interest over three months (91.25 days) and then extrapolated to one year. Individuals are asked to choose between option A and option B for each of the 15 payoff alternatives. One of these alternatives will be randomly selected for the final payoff.

**Table S10.** Payoff matrix for the measurement of social preference.

| | Option A | | Option B | | Preferred payment option (A or B) |
|---|---|---|---|---|---|
| | Person A (INR) | Person B (INR) | Person A (INR) | Person B (INR) | |
| 1 | 100 | 0 | 0 | 0 | A O B |
| 2 | 100 | 0 | 10 | 10 | A O B |
| 3 | 100 | 0 | 20 | 20 | A O B |
| 4 | 100 | 0 | 30 | 30 | A O B |
| 5 | 100 | 0 | 40 | 40 | A O B |
| 6 | 100 | 0 | 50 | 50 | A O B |
| 7 | 100 | 0 | 60 | 60 | A O B |
| 8 | 100 | 0 | 70 | 70 | A O B |
| 9 | 100 | 0 | 80 | 80 | A O B |
| 10 | 100 | 0 | 90 | 90 | A O B |
| 11 | 100 | 0 | 100 | 100 | A O B |

Notes: Individuals are asked to choose between option A and option B for each of the 11 payoff alternatives. Person A refers to the individual making the decision. while Person B is another person randomly selected. One of these alternatives will be randomly selected for the final payoff.

## SI References

1. Lauderdale, D. S., Knutson, K. L., Yan, L. L., Liu, K. & Rathouz, P. J. Self-Reported and Measured Sleep Duration: How Similar Are They? *Epidemiology* **19**, 838–845 (2008).
2. Bessone, P., Rao, G., Schilbach, F., Schofield, H. & Toma, M. The Economic Consequences of Increasing Sleep Among the Urban Poor. *The Quarterly Journal of Economics* **136**, 1887–1941 (2021).
3. Martin Bland, J. & Altman, DouglasG. Statistical methods for assessing agreement between two methods of clinical measurement. *The Lancet* **327**, 307–310 (1986).
